# Supplementary material for: Bis-hydroxylation of Homocitrulline Catalyzed by a Multinuclear Nonheme Iron-Dependent Oxidative Enzyme during RiPP Biosynthesis
Source: bioRxiv. 2026 May 5:2026.05.01.722236. Preprint. [Version 1] doi: 10.64898/2026.05.01.722236 (PMC13174530; doi:10.64898/2026.05.01.722236)
Supplement: Supplement 1 [file media-1.pdf]

SUPPORTING INFORMATION

Bis-hydroxylation of Homocitrulline Catalyzed by a Multinuclear Nonheme Iron-Dependent Oxidative Enzyme during RiPP Biosynthesis

Dayna P. Hebron,[1*] Tucker Shriver,[2] Joshua J. Ziarek,[2] and Amy C. Rosenzweig[1*]

[1]Departments of Molecular Biosciences and of Chemistry, Northwestern University, Evanston, Illinois 60208, United States
[2]Departments of Pharmacology, Feinberg School of Medicine, Northwestern University, Chicago, Illinois 60611, United States
*Corresponding authors: Dayna P. Hebron; Email: dayna.patterson@northwestern.edu; Amy C. Rosenzweig; Email: amyr@northwestern.edu.

Figures S1-S14
Tables S1-S10
DNA and amino acid sequences

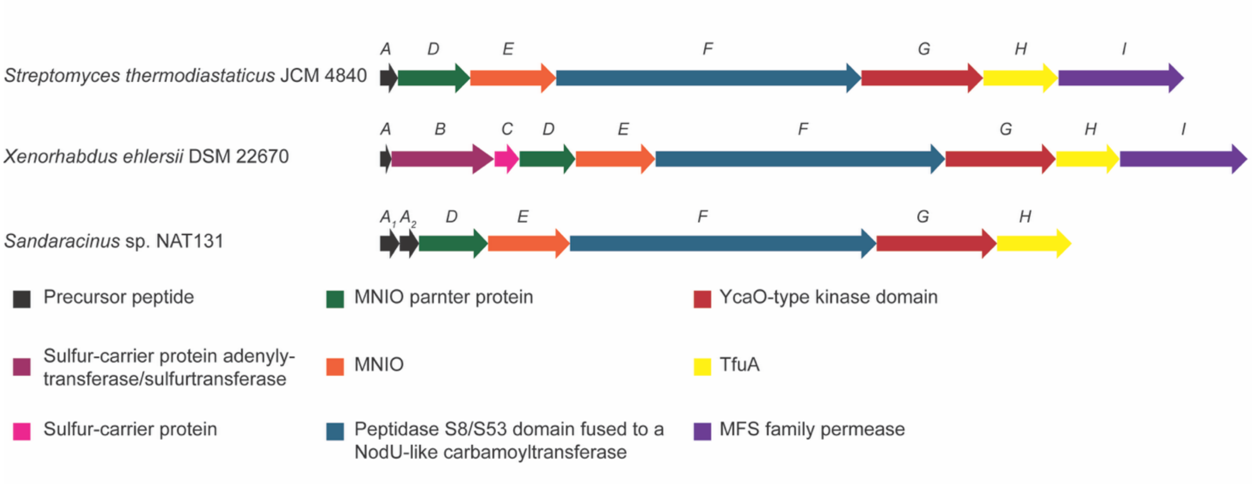

Figure S1. Representative BGCs from Actinomycetota, Pseudomonadota, and Myxococcota phyla.

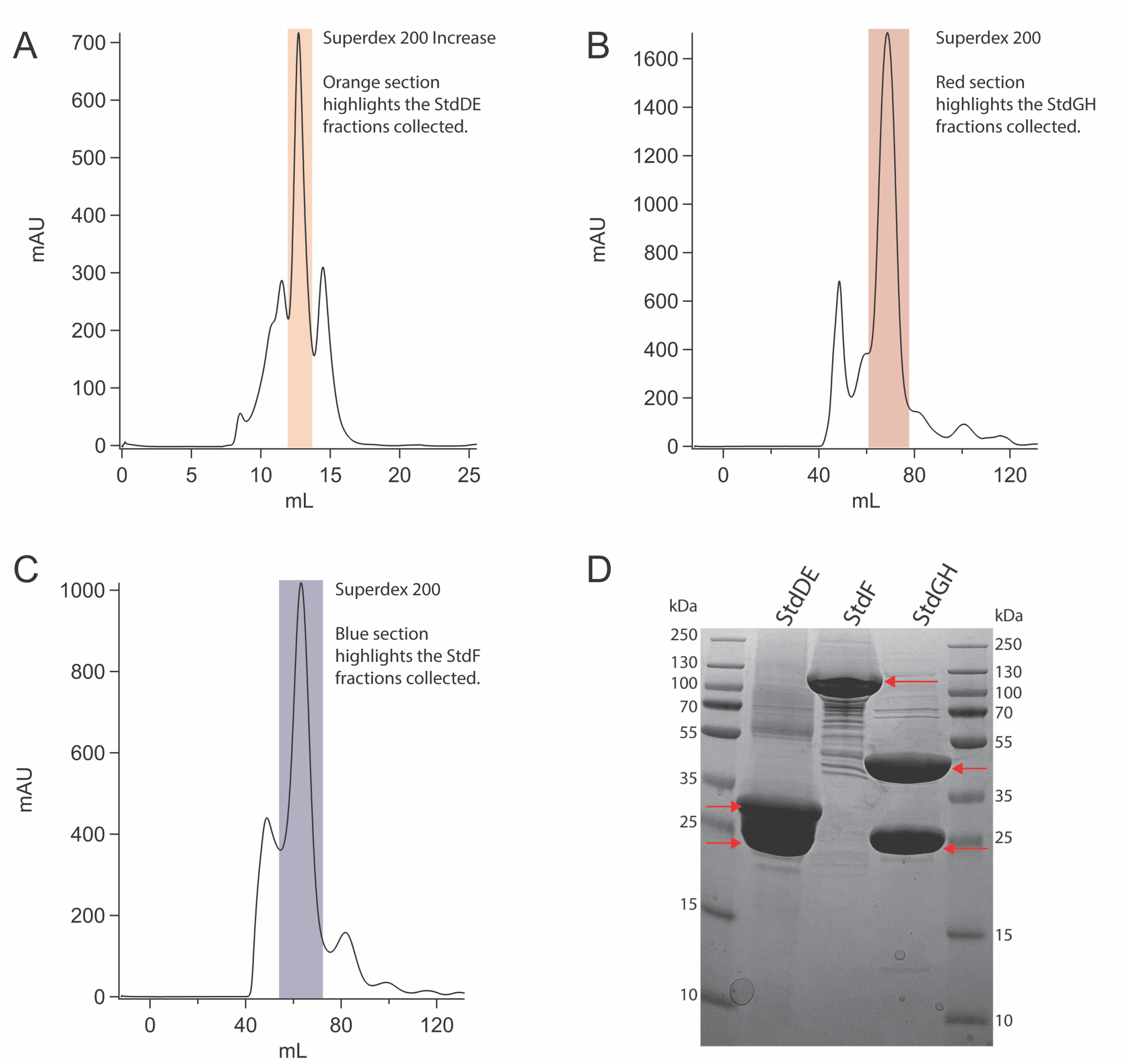

Figure S2. Purification of StdGH, StdDE, and StdF. SEC trace of (A) StdDE, (B) StdGH, and (C) StdF. (D) SDS-PAGE analysis of purified StdD (28.9 kDa) - StdE (32.7 kDa) complex, StdF (114.5 kDa), and StdG (45.4 kDa) - StdH (28.7 kDa) complex.

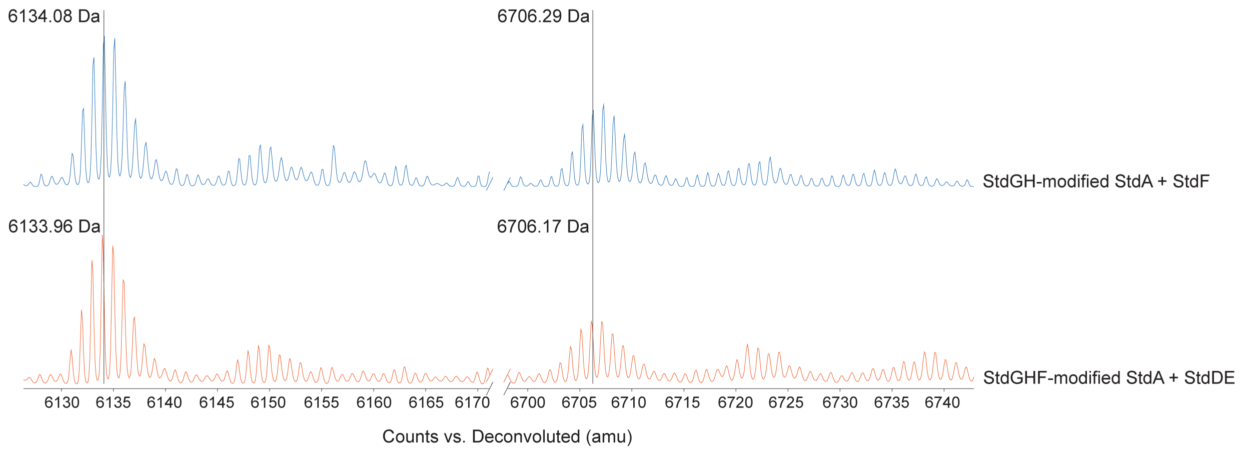

Figure S3. LC-TOF-MS of StdGHF-modified peptide reacted with StdDE, ascorbate, and ammonium iron(II) sulfate hexahydrate.

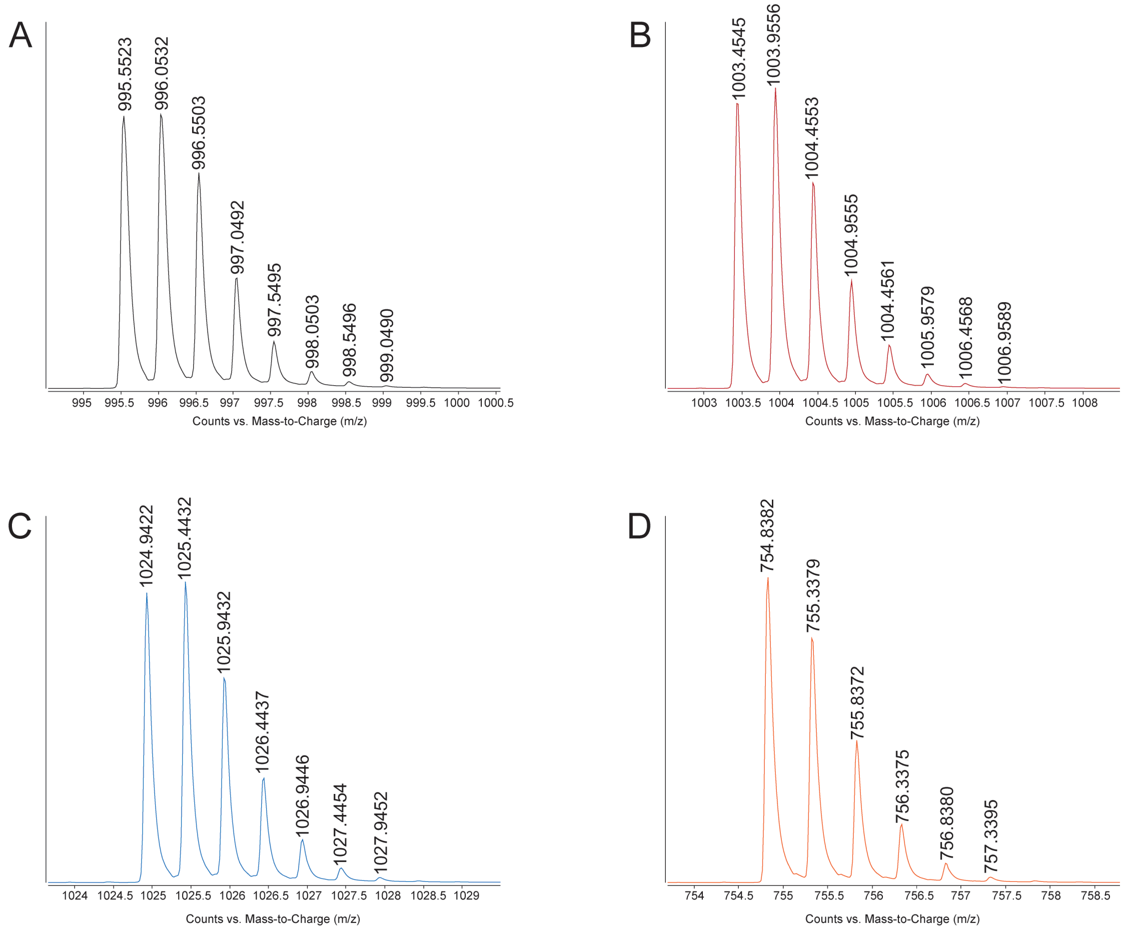

Figure S4. LC-ESI focusing on the $[M+2H]^{2+}$ ion of the Arg-C Ultra fragments of (A) unmodified StdA, (B) StdGH-modified StdA, (C) StdGHF-modified StdA, and (D) StdGHFDE-modified StdA peptides.

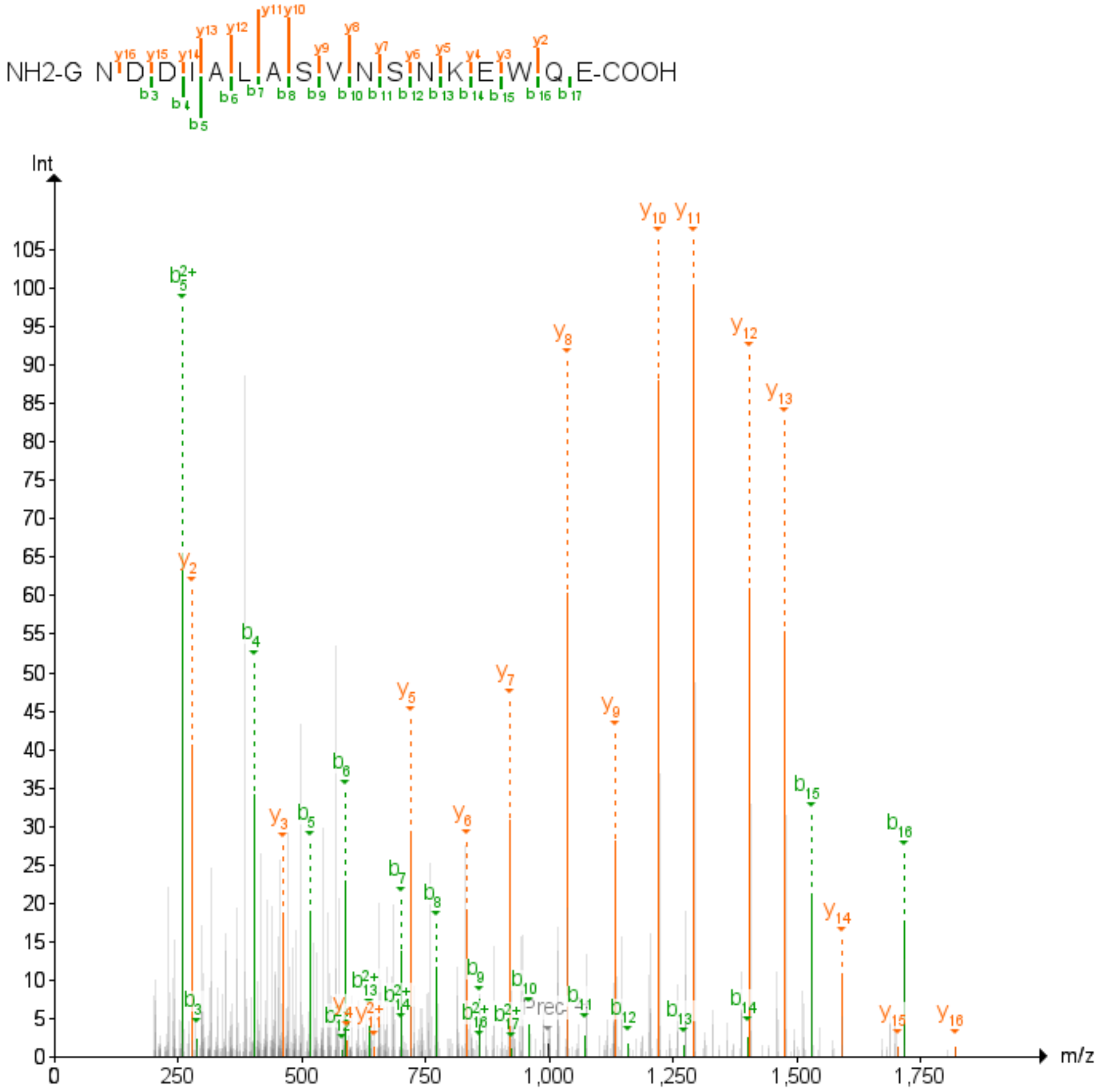

Figure S5. HR-MS/MS spectra of Arg-C Ultra digested unmodified StdA.

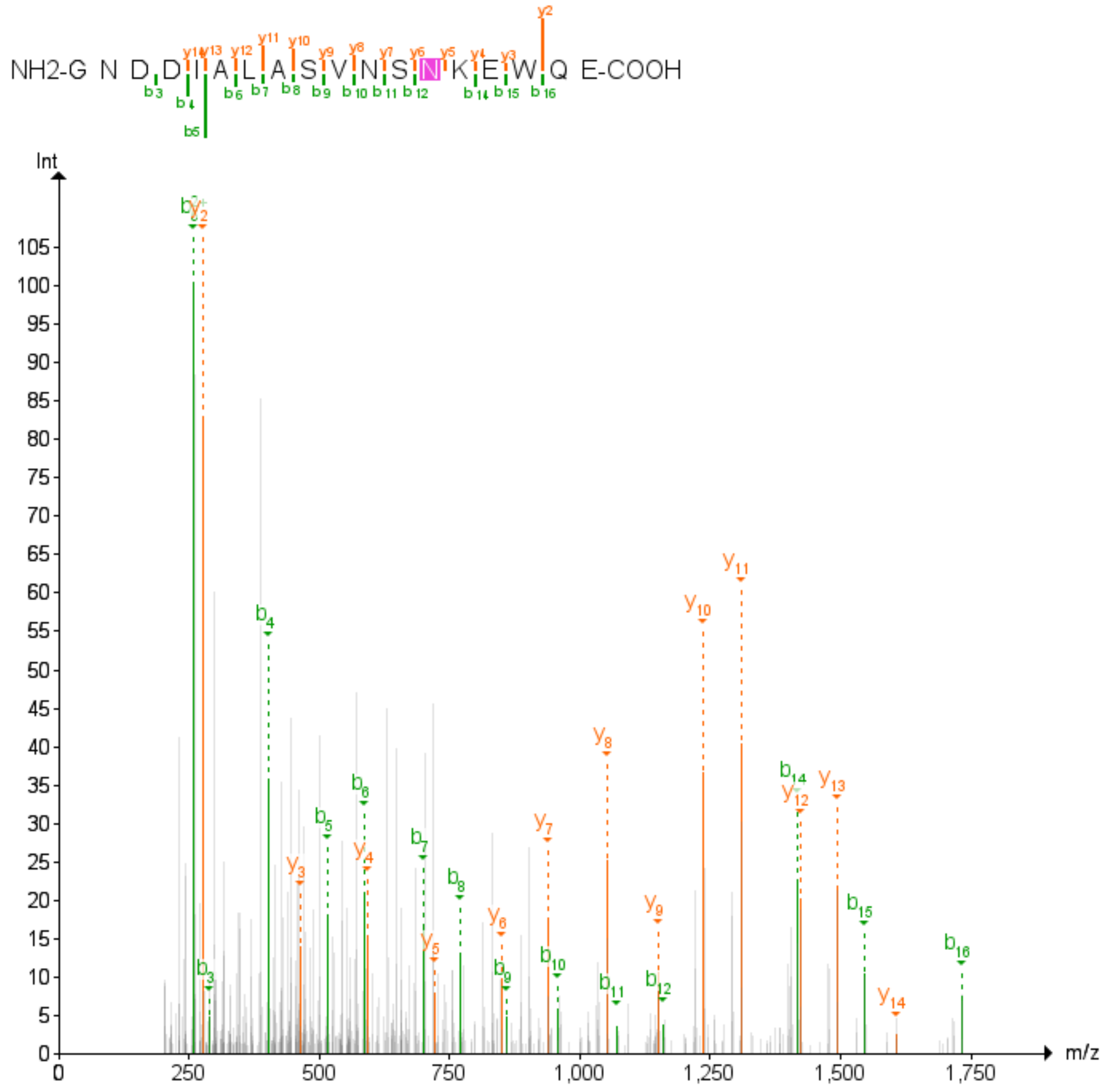

Figure S6. HR-MS/MS spectra of Arg-C Ultra digested StdA peptide following the in vitro reaction with StdGH in the presence of ATP and Na_2S.

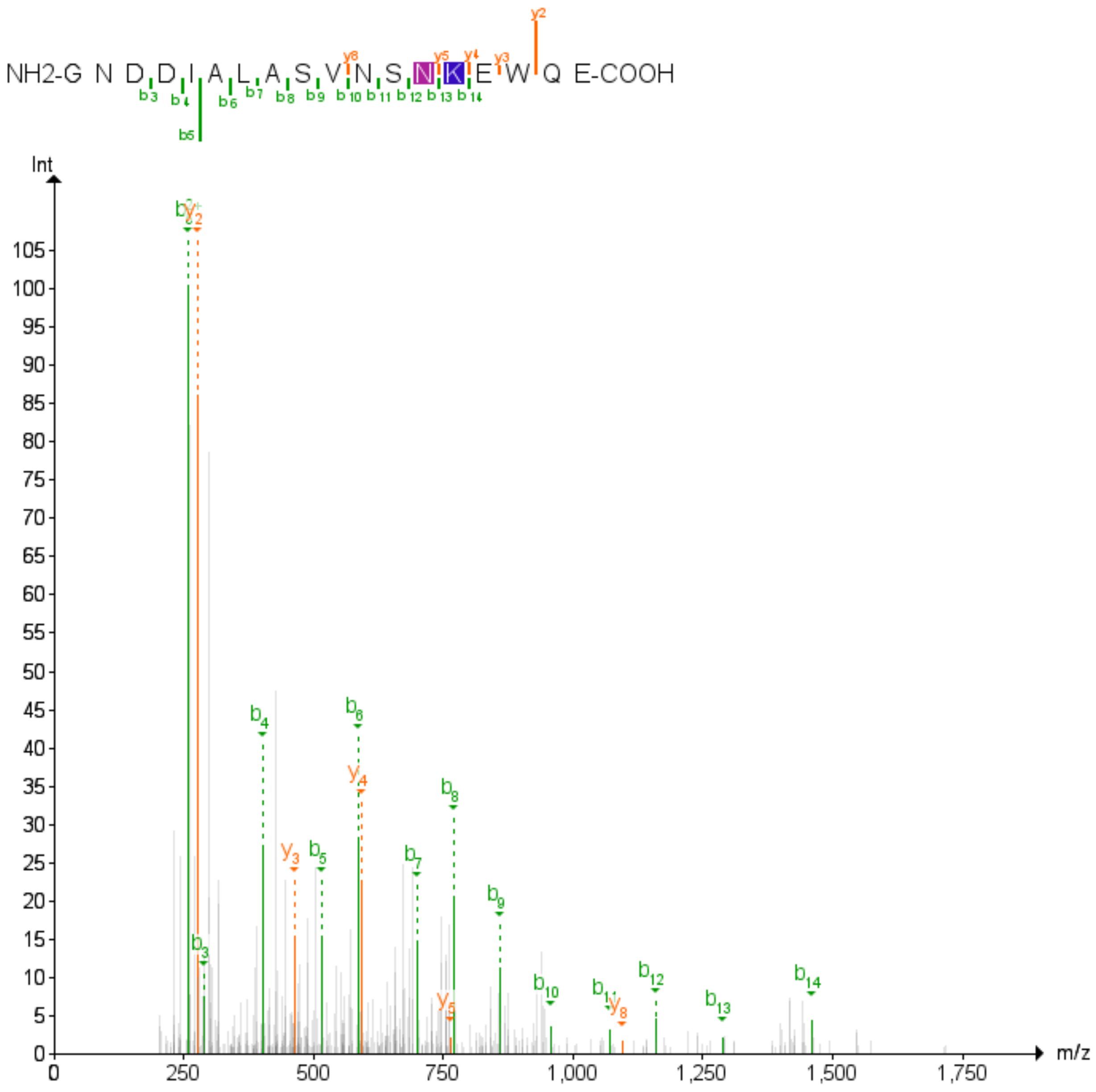

Figure S7. HR-MS/MS spectra for Arg-C Ultra digested for StdGH-modified StdA peptide following the in vitro reaction with StdF in the presence of carbamoyl phosphate and ATP. This species represents the full-length peptide.

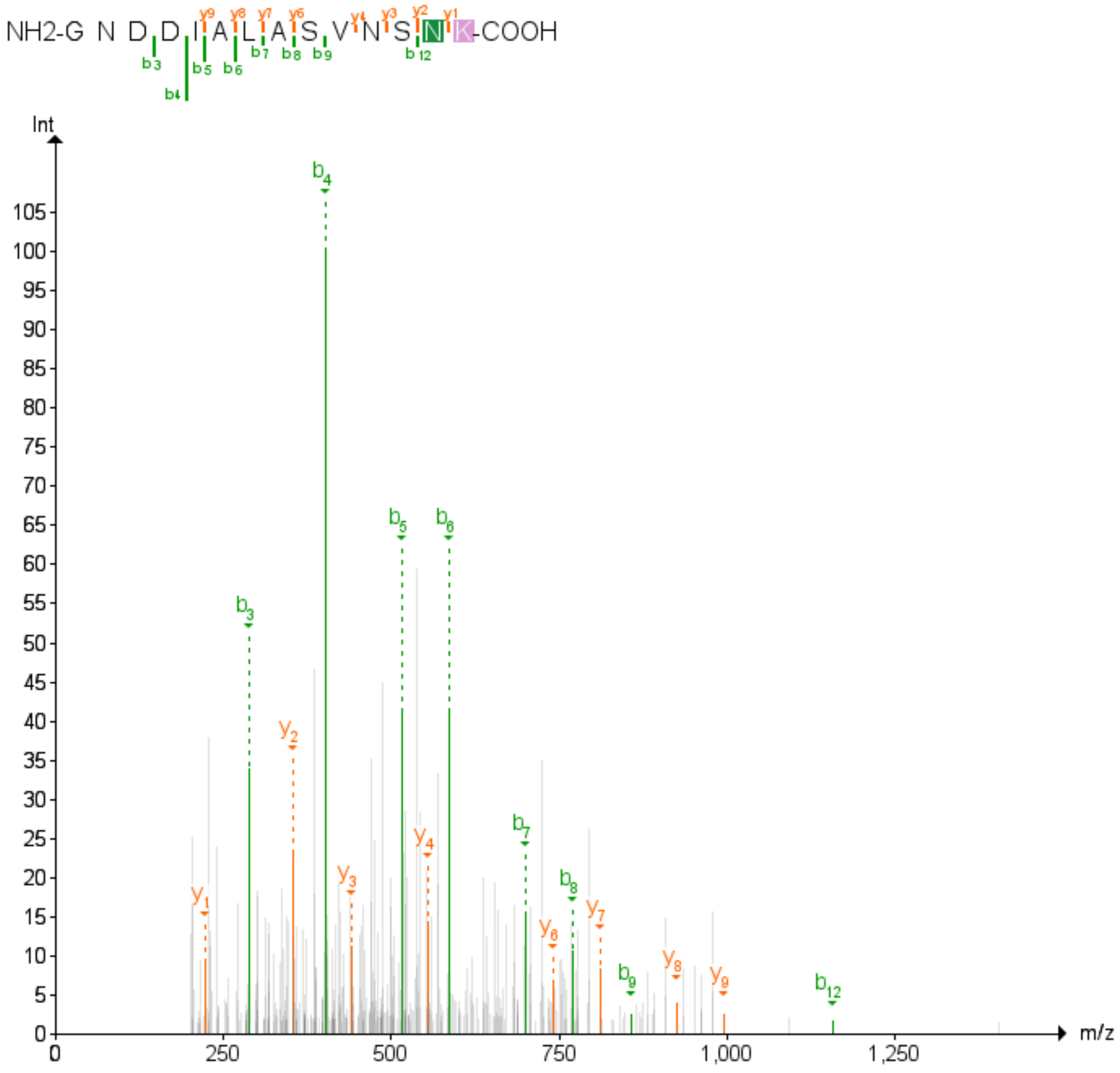

Figure S8. HR-MS/MS spectra for Arg-C Ultra digested for cleaved StdGH-modified StdA peptide following the in vitro reaction with StdF in the presence of carbamoyl phosphate and ATP, and StdDE in the presence of iron and ascorbate.

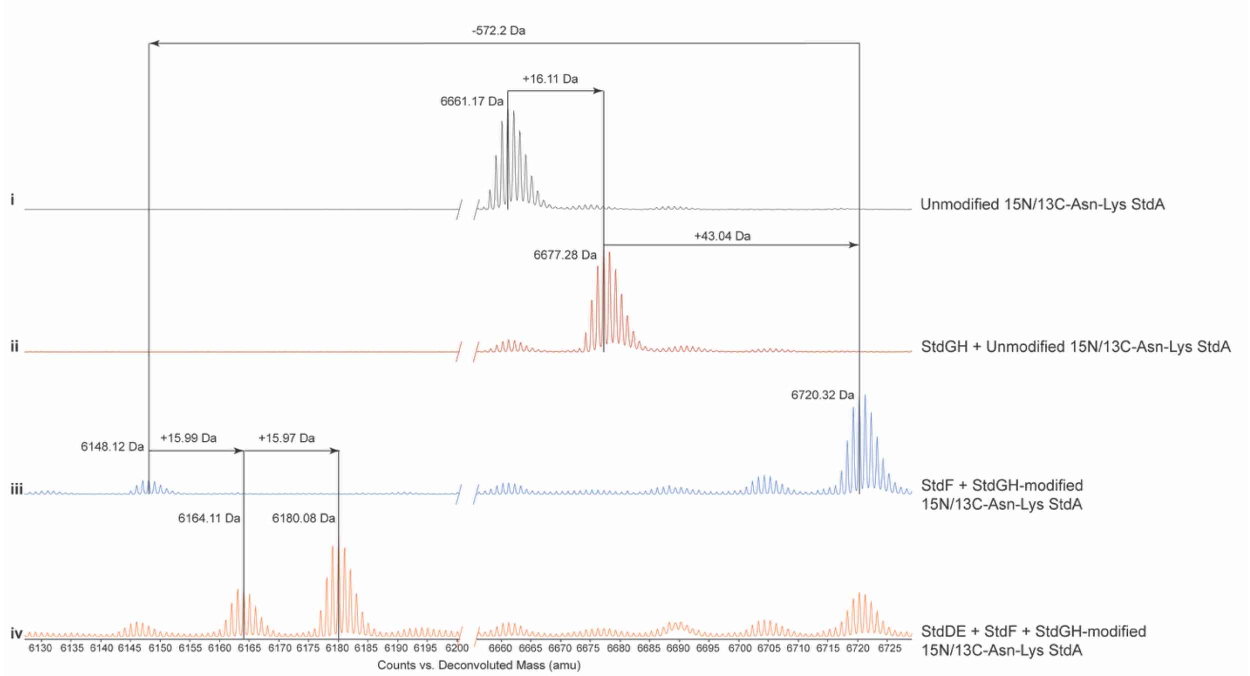

Figure S9. In vitro reactions of StdGH, StdF, and StdDE with $^{13}C/^{15}N$-Asn56-Lys57-StdA monitored by LC-TOF-MS.

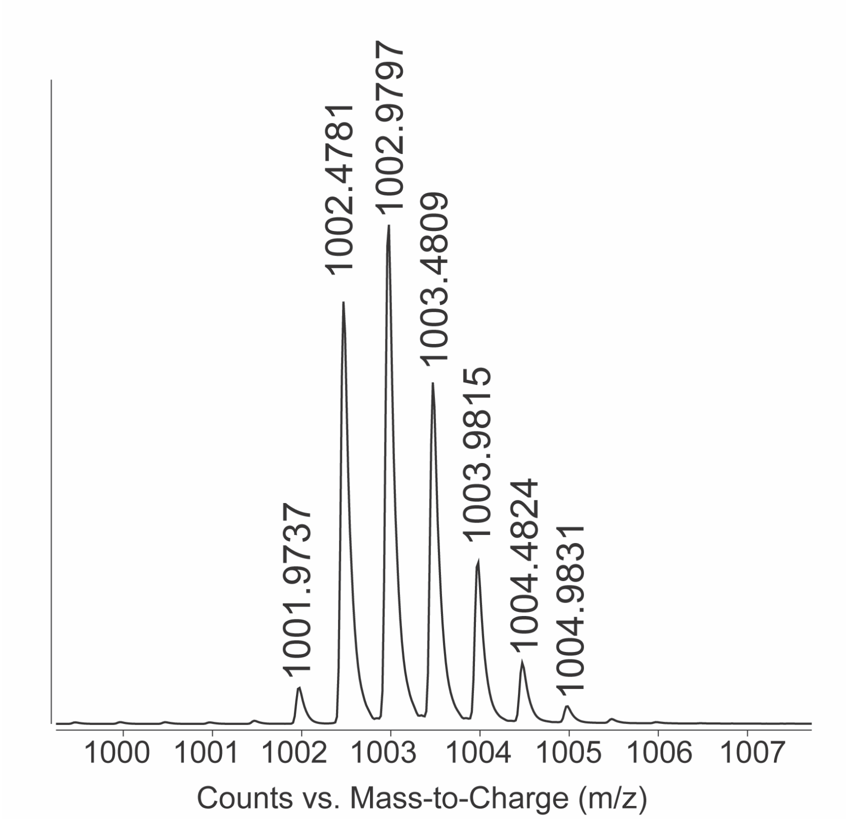

Figure S10. LC-ESI focusing on the $[M+2H]^{2+}$ ion of Arg-C Ultra fragment derived from the 6661.17 Da unmodified $^{15}N/^{13}C$-Asn13-Lys14-StdA.

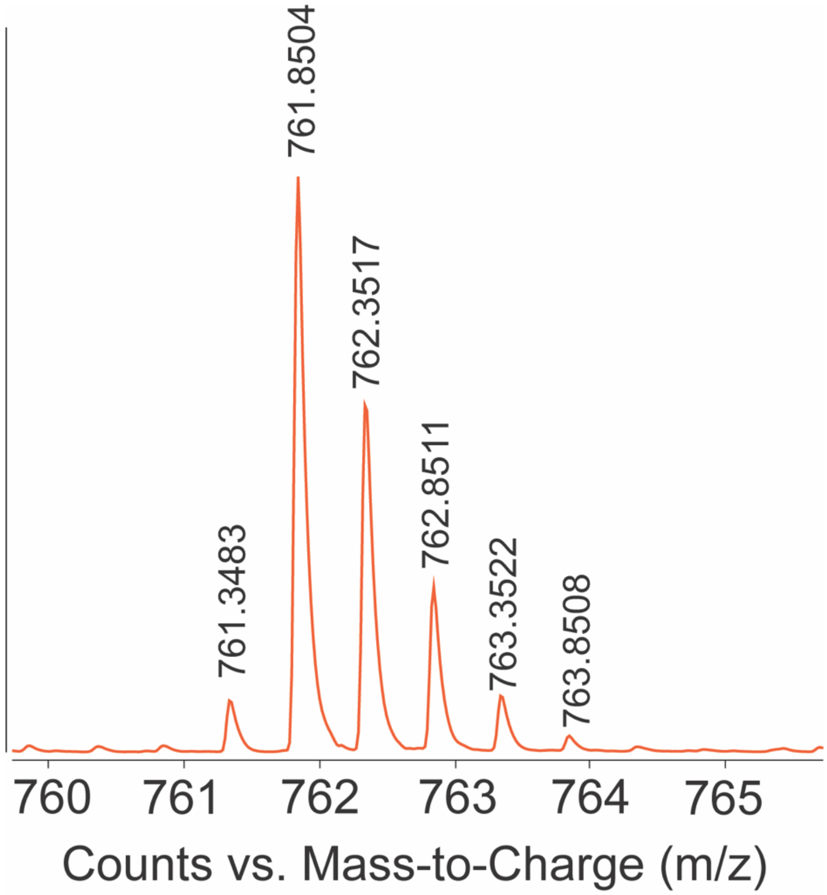

Figure S11. LC-ESI focusing on the $[M+2H]^{2+}$ ion of Arg-C Ultra fragment derived from the 6180.08 Da StdGHFDE-modified $^{15}N/^{13}C$-Asn13-Lys14-StdA.

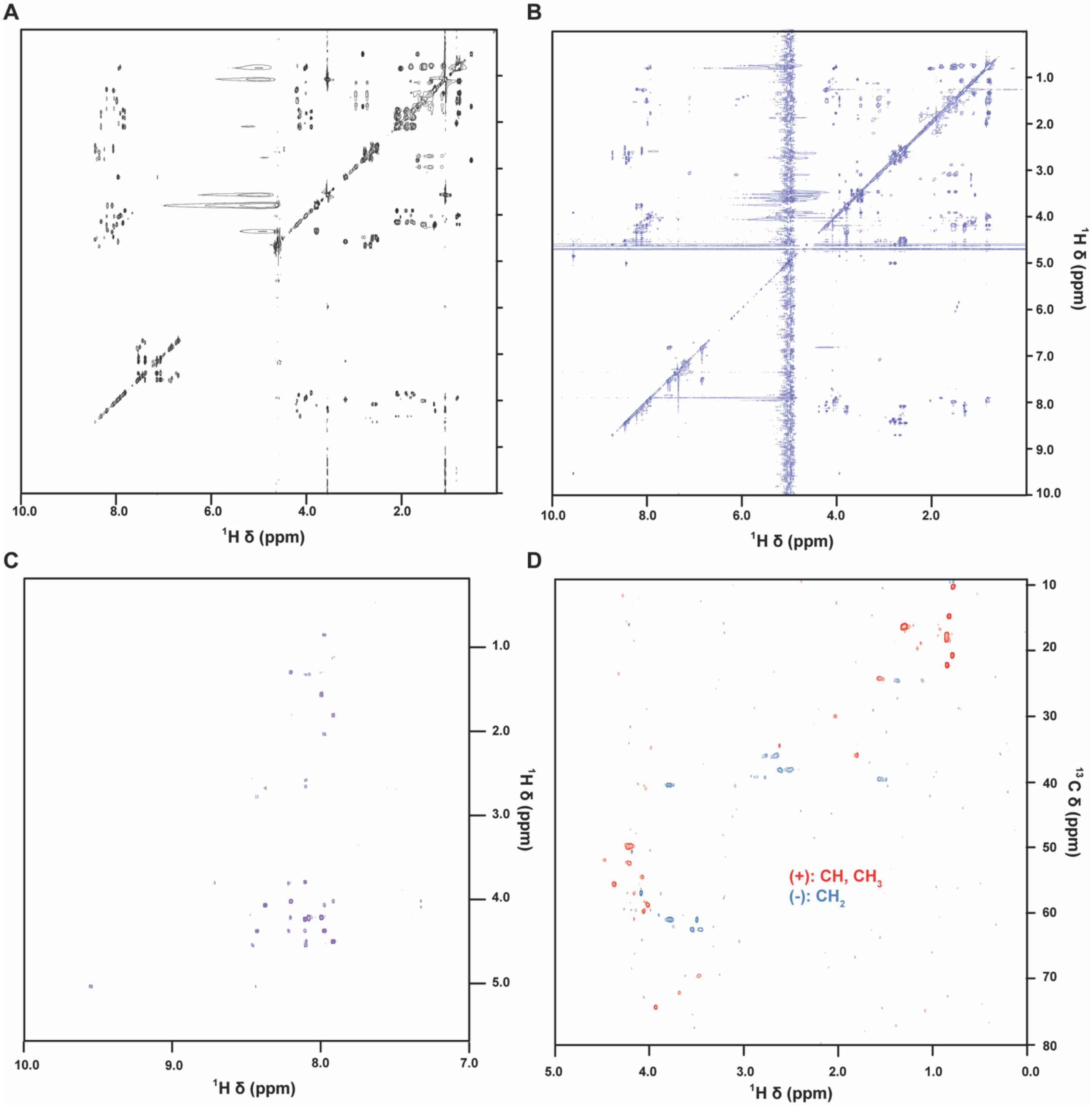

Figure S12. Assignment of Arg-C Ultra digested StdGHFDE-modified ^{15}N/^{13}C-Asn13-Lys14-StdA. (A) TOCSY spectrum of unmodified ^{15}N/^{13}C-Asn13-Lys14-StdA. (B) TOCSY spectrum of modified StdA. (C) HN- contacts in StdGHFDE-modified ^{15}N/^{13}C-Asn13-Lys14-StdA ROESY spectrum with 200 ms mixing time. (D) Multiplicity-edited ^{13}C-HSQC spectrum.

^{1}GNDDIALAS^{10}VNSN*K*

^{1}Gly

C^{α}: 40.5 $H^{\alpha1}$: 3.68 $H^{\alpha2}$: 3.65

^{2}Asn

H^{N}: 8.59 H^{α}: 4.50 $H^{\beta1}$: 2.64

C^{α}: 54.0 C^{β}: 35.9 $H^{\beta2}$: 2.53

^{3}Asp

H^{N}: 8.33 H^{α}: 4.40 $H^{\beta1}$: 2.50

C^{α}: 51.7 C^{β}: 38.3 $H^{\beta2}$: 2.37

^{4}Asp

H^{N}: 7.97 H^{α}: 4.35 $H^{\beta1}$: 2.48

C^{α}: 51.9 C^{β}: 38.0 $H^{\beta2}$: 2.41

^{5}Ile

H^{N}: 7.79 $C^{\gamma1}$: 24.6 $H^{\gamma21}$: 0.700

C^{α}: 58.7 $H^{\gamma11}$: 1.243 $H^{\gamma22}$: 0.689

H^{α}: 3.89 $H^{\gamma12}$: 0.981 C^{δ}: 10.2

C^{β}: 35.9 $C^{\gamma2}$: 14.7 H^{δ}: 0.656

H^{β}: 1.67

^{6}Ala

H^{N}: 7.95 H^{α}: 4.10 $H^{\beta1}$: 1.20

C^{α}: 52.4 C^{β}: 16.4 $H^{\beta2}$: 1.19

^{7}Leu

H^{N}: 7.87 $H^{\beta2}$: 1.36 $H^{\delta12}$: 0.714

C^{α}: 52.4 C^{γ}: 24.2 $C^{\delta2}$: 20.7

H^{α}: 4.08 H^{γ}: 1.44 $H^{\delta21}$: 0.666

C^{β}: 39.6 $C^{\delta1}$: 22.2 $H^{\delta22}$: 0.654

$H^{\beta1}$: 1.40 $H^{\delta11}$: 0.722

^{8}Ala

H^{N}: 8.07 H^{α}: 4.09 $H^{\beta1}$: 1.17

C^{α}: 49.8 C^{β}: 16.2 $H^{\beta2}$: 1.16

^{9}Ser

H^{N}: 7.98 H^{α}: 4.24 H^{β}: 3.66

C^{α}: 55.4 C^{β}: 60.928

^{10}Val

H^{N}: 7.85 H^{β}: 1.90 $C^{\gamma2}$: 17.6

C^{α}: 59.7 $C^{\gamma1}$: 18.3 $H^{\gamma21}$: 0.732

H^{α}: 3.93 $H^{\gamma11}$: 0.728 $H^{\gamma22}$: 0.717

C^{β}: 30.0 $H^{\gamma12}$: 0.714

^{11}Asn

H^{N}: 8.25 H^{α}: 4.58 $H^{\beta1}$: 2.67

C^{α}: 55.1 C^{β}: 36.1 $H^{\beta2}$: 2.55

^{12}Ser

H^{N}: 8.04 H^{α}: 4.26 H^{β}: 3.66

C^{α}: 55.6 C^{β}: 60.930

^{13}Modified-Asn

H^{N}: 8.33 H^{α}: 4.90 C^{γ}: 174.3

N: 125.1 C^{β}: 39.3 N^{δ}: 113.4

C^{S}: 171.5 $H^{\beta1}$: 2.76 $H^{\delta1}$: 7.40

C^{α}: 56.1 $H^{\beta2}$: 2.65 $H^{\delta2}$: 6.75

^{13}Unmodified-Asn

H^{N}: 8.26 H^{α}: 4.34 C^{γ}: 174.3

N: 121.3 C^{β}: 38.2 N^{δ}: 113.4

C^{O}: 172.4 $H^{\beta1}$: 2.59 $H^{\delta1}$: 7.40

C^{α}: 52.8 $H^{\beta2}$: 2.50 $H^{\delta2}$: 6.75

^{14}Modified-Lys

H^{N}: 9.43 C^{γ}: 69.5 H^{ε}: 3.00

N: 112.2 H^{γ}: 3.35 N^{ζ}: 87.0

C^{α}: 62.5 C^{δ}: 32.5 H^{ζ}: 6.00

H^{α}: 4.74 $H^{\delta1}$: 1.52 C^{η}: 171.4

C^{β}: 74.3 $H^{\delta2}$: 1.36 N^{θ}: 117.5

H^{β}: 3.81 C^{ε}: 45.3 H^{θ}: 7.65

^{14}Unmodified-Lys

H^{N}: 8.06 H^{β}: 1.51 C^{δ}: 21.8

N: 112.2 C^{γ}: 30.2 H^{δ}: 121.1

C^{α}: 53.7 $H^{\gamma1}$: 1.62 C^{ε}: 39.4

H^{α}: 4.13 $H^{\gamma2}$: 1.58 H^{ε}: 2.83

C^{β}: 26.3

Figure S13. Arg-C Ultra digested StdGHFDE-modified $^{15}N/^{13}C$-Asn13-Lys14-StdA chemical shift list. Complete chemical shift assignments for the StdGHFDE-modified $^{15}N/^{13}C$-Asn13-Lys14-StdA and unmodified $^{15}N/^{13}C$-Asn13-Lys14-StdA Asn13 and Lys14 residues. Amino acid sequence shown at top with sites of modification indicated by an asterisk.

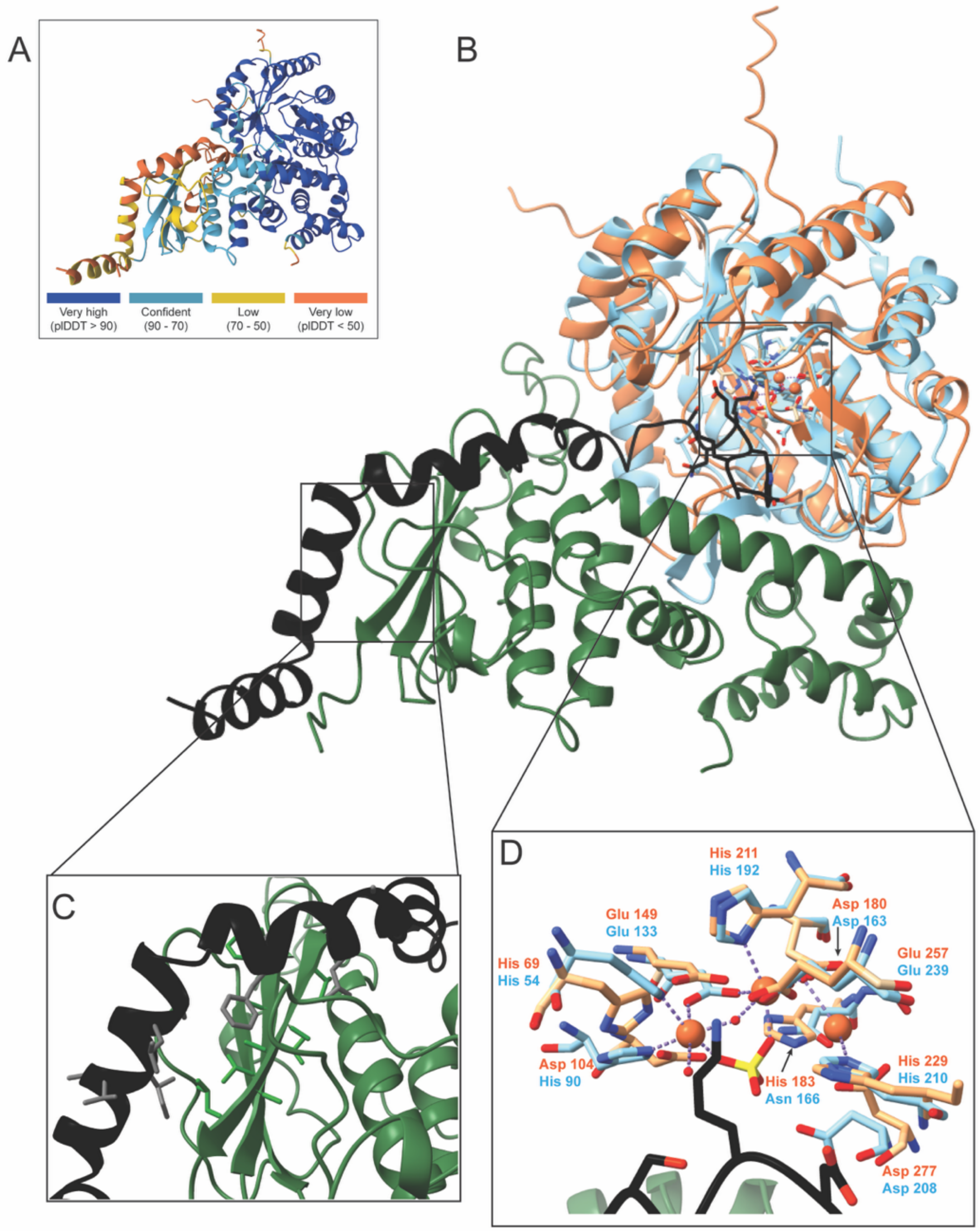

Figure S14. AlphaFold 3 model of StdDE in complex with cleaved StdA. (A) Confidence metrics for the AlphaFold 3-predicted structure. Per-residue pLDDT scores are shown using the color scale indicated in the legend, with higher values indicating higher confidence. (B) AlphaFold 3-predicted model of the StdDE–StdA complex showing StdA (black), StdD (green), and StdE (orange). StdE is superimposed with the crystal structure of MbnB from *Methylosinus trichosporium* OB3b (blue; PDB ID: 7TCR). (C) Close-up view of the predicted RRE site in StdD (green), highlighting hydrophobic residues interacting with hydrophobic residues in the StdA leader peptide (black). (D) Conserved residues in StdE are predicted to coordinate Fe ions and modify StdA Lys57, based on comparison with the MbnB crystal structure.

Table S1. IMG Gene ID, gene names, and predicted functions of genes in the *std* cluster.

Gene ID	Gene name	Predicted function
8109937978	*stdA*	Precursor peptide
8109937979	*stdD*	MNIO partner protein
8109937980	*stdE*	MNIO
8109937981	*stdF*	Peptidase S8/S53 domain fused to a NodU-like carbamoyltransferase
8109937982	*stdG*	YcaO-type kinase domain
8109937983	*stdH*	TfuA
8109937984	*stdI*	MFS family permease

Table S2. Prevalence of StdE in microbial genomes. IMG Gene ID and strains are provided. Entries are in order from the highest sequence identity (%ID) to the StdD sequence from *S. thermodiastaticus* JCM 4840. Precursor sequences were identified through a manual search of the genome's region upstream of each StdE homolog.

Gene ID	Strain	Phylum	%ID (MNIO/StdE)	Precursor peptide sequence
8109937980	*Streptomyces thermodiastaticus* JCM 4840	Actinomycetota	299/299 100%	MSDNTAAVLSAIDSDKAL RSAVIRHLATTHAHEFVG PLRAEFRGNDDIALASVN SNKEWQE
2951696985	*Actinomycetota* bacterium 052637	Actinomycetota	295/299 i98.66%	MSDNTAAVLSAIDSDKAL RSAVIRHLATTHAHEFVG PLRAEFRGNDDIALASVN SNKEWQE
8112178169	*Streptomyces thermoviolaceus apingens* JCM 4312	Actinomycetota	295/299 98.66%	MSDNTAAVLSAIDSDKAL RSAVIRHLATTHAHEFVG PLRAEFRGNDDIALASVN SNKEWQE
8112201871	*Streptomyces thermoviolaceus thermoviolaceus* JCM 4843	Actinomycetota	295/299 98.66%	MSDNTAAVLSAIDSDKAL RSAVIRHLATTHAHEFVG PLRAEFRGNDDIALASVN SNKEWQE
2866522241	*Streptomyces* sp. WAC00469	Actinomycetota	295/299 98.66%	MSDNTAAVLSAIDSDKAL RSAVIRHLATTHAHEFVG PLRAEFRGNDDIALASVN SNKEWQE
2940111087	*Actinomycetota* bacterium 52594	Actinomycetota	295/299 98.66%	MSDNTAAVLSAIDSDKAL RSAVIRHLATTHAHEFVG PLRAEFRGNDDIALASVN SNKEWQE
2896974795	*Streptomyces* sp. DSM 40868	Actinomycetota	278/299 92.98%	MSDNTAAVLSAIDSDKAL RSAVIRHLATTHANEFVS PLRAEFRGNDDIALASVN SNKEWQE
2944895943	*Streptomyces* sp. 25648	Actinomycetota	278/299 92.98%	MSDNTAAVLSAIDSDKAL RSAVIRHLATTHANEFVS PLRAEFRGNDDIALASVN SNKEWQE
8012369728	*Actinomycetota* bacterium 017043_259_D4	Actinomycetota	278/299 92.98%	MSDNTAAVLSAIDSDKAL RSAVIRHLATTHANEFVS PLRAEFRGNDDIALASVN SNKEWQE
2975123176	*Actinomycetota* bacterium 21046_187_C2	Actinomycetota	278/299 92.98%	MSDNTAAVLSAIDSDKAL RSAVIRHLATTHANEFVS PLRAEFRGNDDIALASVN SNKEWQE
2960294871	*Actinomycetota* bacterium 21112	Actinomycetota	278/299 92.98%	MSDNTAAVLSAIDSDKAL RSAVIRHLATTHANEFVS PLRAEFRGNDDIALASVN SNKEWQE
2974034638	*Actinomycetota* bacterium 22290	Actinomycetota	278/299 92.98%	MSDNTAAVLSAIDSDKAL RSAVIRHLATTHANEFVS PLRAEFRGNDDIALASVN SNKEWQE
2766392705	*Streptomyces lavenduligriseus* NRRL ISP-5487	Actinomycetota	278/299 92.98%	MSDNTAAVLSAIDSDKAL RSAVIRHLATTHANEFVS PLRAEFRGNDDIALASVN SNKEWQE

2945369264	Actinomycetota bacterium 001328	Actinomycetota	278/299 92.98%	MSDKTAAVLSAIDSDKAL RSAVIRHLATTHANEFVS PLRAEFRGNDDIALASVN SNKEWQE
8113491221	*Streptomyces eurythermus* JCM 4206	Actinomycetota	275/299 91.97%	MSDNTAAVLSAIDSDKAL RSAVIRHLATTHANEFVS PLRAEFRGNDDIALASVN SNKEWQE
8012981714	*Actinomycetota* bacterium 014880_285_H06	Actinomycetota	275/299 91.97%	MSDNTAAVLSAIDSDKAL RSAVIRHLATTHANEFVS PLRAEFRGNDDIALASVN SNKEWQE
8012350675	*Actinomycetota* bacterium 014664_259_C8	Actinomycetota	275/299 91.97%	MSDNTAAVLSAIDSDKAL RSAVIRHLATTHANEFVS PLRAEFRGNDDIALASVN SNKEWQE
8015150593	*Actinomycetota* bacterium 014665_263_C06	Actinomycetota	275/299 91.97%	MSDNTAAVLSAIDSDKAL RSAVIRHLATTHANEFVS PLRAEFRGNDDIALASVN SNKEWQE
8056831951	*Streptomyces barringtoniae* JA03	Actinomycetota	238/299 79.60%	MSDKTTAILAALDGDKTL RSAIIQHLATTHAQEFVNP LRAELGGNDDLSLGAVNS NKEWQE
2856761647	*Streptacidiphilus pinicola* MMS16-CNU450	Actinomycetota	202/281 71.89%	MAEINEILAAIDSSKKVRS AVIERLVSTHAQEVVNPL RAIAGDDVSWSSITSNKD WSE
2775746310	*Myxococcales* bacterium CPC88	Myxococcota	131/276 47.46%	MVEALSKKLKGLSFEDLA EVIAEDREFREALIKHIAK DHAEDYLGEVNVNARVL AGLSARVVSSNKEWQE
2775746310	*Myxococcales* bacterium CPC88	Myxococcota	131/276 47.46%	MVLVGSKKLKGISFEEITA LIDEDREFREAMIRLLARD HAEDYLKQVNLNVGALTE LSARVVSSAKEWQD
2775845471	*Sandaracinus* sp. NAT131	Myxococcota	131/276 47.46%	MVEALSKKLKGLSFEDLA EVIAEDREFREALIKHIAK DHAEDYLGEVNVNARVL AGLSARVVSSNKEWQE
2775845471	*Sandaracinus* sp. NAT131	Myxococcota	131/276 47.46%	MVLVGSKKLKGISFEEITA LIDEDREFREAMIRLLARD HAEDYLKQVNLNVGALTE LSARVVSSAKEWQD
2775851308	*Sandaracinus* sp. SP2993	Myxococcota	131/276 47.46%	MVEALSKKLKGLSFEDLA EVIAEDREFREALIKHIAK DHAEDYLGEVNVNARVL AGLSARVVSSNKEWQE
2775851308	*Sandaracinus* sp. SP2993	Myxococcota	131/276 47.46%	MVLVGSKKLKGISFEEITA LIDEDREFREAMIRLLARD HAEDYLKQVNLNVGALTE LSARVVSSAKEWQD
2982850127	*Dickeya fangzhongdai* M074	Pseudomonadota	121/276 43.84%	MDKAQELELAVAELNAEN TEAENEILLDVTCMVSNK EWQE
2982845965	*Dickeya fangzhongdai* M005	Pseudomonadota	121/276 43.84%	MDKAQELELAVAELNAEN TEAENEILLDVTCMVSNK EWQE
2630173480	*Dickeya fangzhongdai* ND14b	Pseudomonadota	121/276 43.84%	MDKAQELELAVAELNAEN TEAENEILLDVTCMVSNK EWQE

2776528989	*Dickeya fangzhongdai* DSM 101947	Pseudomonadota	121/277 43.68%	MDKAQELELAVAELNAEN TEAENEILLDVTCMVSNK EWQE
8113173844	*Dickeya fangzhongdai* CGMCC 1.15464	Pseudomonadota	121/277 43.68%	MDKAQELELAVAELNAEN TEAENEILLDVTCMVSNK EWQE
2860871481	*Pseudomonas* sp. R1-43-08	Pseudomonadota	122/280 43.57%	MELQEKDVALEEQELAIA KSEELSEELVLSLAHHAS DKEWQE
2651807005	*Dickeya fangzhongdai* B16	Pseudomonadota	120/276 43.48%	MDKAQELELAVAELNAEN TEAENEILLDVTCMVSNK EWQE
2559184145	*Dickeya zeae* NCPPB 3531	Pseudomonadota	120/276 43.48%	MDKAQELELAVAELNAEN TEAENEILLDVTCMVSNK EWQE
2654976169	*Dickeya fangzhongdai* S1	Pseudomonadota	120/276 43.48%	MDKAQELELAVAELNAEN TEAENEILLDVTCMVSNK EWQE
2814988251	*Dickeya dadantii* Secpp1600	Pseudomonadota	120/276 43.48%	MDKAQELELAVAELNAEN TEAENEILLDVTCMVSNK EWQE
2831161822	*Dickeya zeae* A5272	Pseudomonadota	120/276 43.48%	MDKAQELELAVAELNAEN TEAENEILLDVTCMVSNK EWQE
2982856433	*Dickeya zeae* A5272	Pseudomonadota	120/276 43.48%	MDKAQELELAVAELNAEN TEAENEILLDVTCMVSNK EWQE
2847241304	*Dickeya fangzhongdai* PA1	Pseudomonadota	120/276 43.48%	MDKAQELELAVAELNAEN TEAENEILLDVTCMVSNK EWQE
2559208148	*Dickeya* sp. MK7	Pseudomonadota	120/276 43.48%	MDKAQELELAVAELNAEN TEAENEILLDVTCMVSNK EWQE
2982835375	*Dickeya zeae* A5410	Pseudomonadota	119/276 43.12%	MDKAQELELAVAELNAEN AEAENEILLDVTCTVSNKE WQE
2559192607	*Dickeya zeae* MK19	Pseudomonadota	119/276 43.12%	MDKAQELELAVAELNAEN AEAENEILLDVTCTVSNKE WQE
2599197678	*Dickeya zeae* NCPPB 3532	Pseudomonadota	119/276 43.12%	MDKAQELELAVAELNAEN AEAENEILLDVTCTVSNKE WQE
2770339707	*Dickeya zeae* AG740	Pseudomonadota	119/276 43.12%	MDKAQELELAVAELNAEN AEAENEILLDVTCTVSNKE WQE
2843877218	*Dickeya zeae* MS2	Pseudomonadota	119/276 43.12%	MDKAQELELAVAELNAEN AEAENEILLDVTCTVSNKE WQE
2831156921	*Dickeya zeae* A5410	Pseudomonadota	119/276 43.12%	MDKAQELELAVAELNAEN AEAENEILLDVTCTVSNKE WQE
2559179939	*Dickeya zeae* NCPPB 2538	Pseudomonadota	119/276 43.12%	MDKAQELELAVAELNAEN AEAENEILLDVTCTVSNKE WQE
2982838804	*Dickeya zeae* PL65	Pseudomonadota	119/276 43.12%	MDKAQELELAVAELNAEN AEAENEILLDVTCTVSNKE WQE
646448342	*Dickeya parazeae* Ech586	Pseudomonadota	118/276 42.75%	MDKAQELELAVAELNAEN AEAENEILLDVTCTVSNKE WQE

8085543270	*Burkholderia* sp. MS455	Pseudomonadota	119/279 42.65%	MVEQNQFILEEDEKQIDANQVSDEELTLALSSSLSNKEWQE
2552850907	*Dickeya solani* MK10	Pseudomonadota	117/275 42.55%	MDKAQELELAVAELNAENTEAENEILLDVTCMVSNKEWQE
2849505397	*Photorhabdus* sp. HUG-39	Pseudomonadota	117/275 42.55%	MDKAQELELAVAELNAENADVENELLLDVASGVSNKEWQE
2847232563	*Dickeya solani* IFB0223	Pseudomonadota	117/275 42.55%	MDKAQELELAVAELNAENTEAENEILLDVTCMVSNKEWQE
2854530711	*Dickeya solani* IFB_0158	Pseudomonadota	117/275 42.55%	MDKAQELELAVAELNAENTEAENEILLDVTCMVSNKEWQE
2895735474	*Photorhabdus kayaii* C-HU2	Pseudomonadota	117/275 42.55%	MDKAQELELAVAELNAENADVENELLLDVASGVSNKEWQE
2547655134	*Dickeya solani* GBBC 2040	Pseudomonadota	117/275 42.55%	MDKAQELELAVAELNAENTEAENEILLDVTCMVSNKEWQE
2843868778	*Dickeya solani* D s0432-1	Pseudomonadota	117/275 42.55%	MDKAQELELAVAELNAENTEAENEILLDVTCMVSNKEWQE
2847245787	*Dickeya solani* IFB 0099	Pseudomonadota	117/275 42.55%	MDKAQELELAVAELNAENTEAENEILLDVTCMVSNKEWQE
2841397997	*Dickeya solani* RNS 08.23.3.1.A	Pseudomonadota	117/275 42.55%	MDKAQELELAVAELNAENTEAENEILLDVTCMVSNKEWQE
2565704031	*Dickeya solani* D s0432-1	Pseudomonadota	117/275 42.55%	MDKAQELELAVAELNAENTEAENEILLDVTCMVSNKEWQE
2552837950	*Dickeya solani* MK16	Pseudomonadota	117/275 42.55%	MDKAQELELAVAELNAENTEAENEILLDVTCMVSNKEWQE
2895749269	*Photorhabdus kayaii* M-HU2	Pseudomonadota	117/275 42.55%	MDKAQELELAVAELNAENADVENELLLDVASGVSNKEWQE
2684803920	*Dickeya solani* RNS 07.7.3B	Pseudomonadota	117/275 42.55%	MDKAQELELAVAELNAENTEAENEILLDVTCMVSNKEWQE
2854598718	*Dickeya solani* PPO 9019	Pseudomonadota	117/275 42.55%	MDKAQELELAVAELNAENTEAENEILLDVTCMVSNKEWQE
2673345593	*Dickeya solani* PPO 9134	Pseudomonadota	117/275 42.55%	MDKAQELELAVAELNAENTEAENEILLDVTCMVSNKEWQE
2841387782	*Dickeya solani* IPO 2222	Pseudomonadota	117/275 42.55%	MDKAQELELAVAELNAENTEAENEILLDVTCMVSNKEWQE
2854615059	*Dickeya solani* IFB_0221	Pseudomonadota	117/275 42.55%	MDKAQELELAVAELNAENTEAENEILLDVTCMVSNKEWQE
2552833607	*Dickeya* solani IPO 2222	Pseudomonadota	117/275 42.55%	MDKAQELELAVAELNAENTEAENEILLDVTCMVSNKEWQE
2834863738	*Dickeya dianthicola* WV516	Pseudomonadota	117/276 42.39%	MDKAQELELAVAELNAENTESENEILLDVACTVSNKEWQE

2552855238	*Dickeya dianthicola* IPO 980	Pseudomonadota	117/276 42.39%	MDKAQELELAVAELNAEN TESENEILLDVACTVSNKE WQE
2847228062	*Dickeya dianthicola* ME23	Pseudomonadota	117/276 42.39%	MDKAQELELAVAELNAEN TESENEILLDVACTVSNKE WQE
2854576163	*Dickeya dianthicola* DE440	Pseudomonadota	117/276 42.39%	MDKAQELELAVAELNAEN TESENEILLDVACTVSNKE WQE
8079946014	*Dickeya dianthicola* LAR.16.03.LID	Pseudomonadota	117/276 42.39%	MDKAQELELAVAELNAEN TESENEILLDVACTVSNKE WQE
2552859749	*Dickeya dianthicola* NCPPB 3534	Pseudomonadota	117/276 42.39%	MDKAQELELAVAELNAEN TESENEILLDVACTVSNKE WQE
2630773784	*Dickeya dianthicola* RNS04.9	Pseudomonadota	117/276 42.39%	MDKAQELELAVAELNAEN TESENEILLDVACTVSNKE WQE
2552841937	*Dickeya dianthicola* GBBC 2039	Pseudomonadota	117/276 42.39%	MDKAQELELAVAELNAEN TESENEILLDVACTVSNKE WQE
2552846655	*Dickeya dianthicola* NCPPB 453	Pseudomonadota	117/276 42.39%	MDKAQELELAVAELNAEN TESENEILLDVACTVSNKE WQE
2854609977	*Dickeya dianthicola* S4.16.03.P2.4	Pseudomonadota	117/276 42.39%	MDKAQELELAVAELNAEN TESENEILLDVACTVSNKE WQE
2559220795	*Dickeya* sp. CSL RW240	Pseudomonadota	117/276 42.39%	MDKAQELELAVAELNAEN TESENEILLDVACTVSNKE WQE
2854602561	*Dickeya dianthicola* SS70	Pseudomonadota	117/276 42.39%	MDKAQELELAVAELNAEN TESENEILLDVACTVSNKE WQE
2854587708	*Dickeya dianthicola* S4.16.03.LID	Pseudomonadota	117/276 42.39%	MDKAQELELAVAELNAEN TESENEILLDVACTVSNKE WQE
2981433753	*Paraburkholderia* sp. GAS199	Pseudomonadota	117/277 42.24%	MNEEMQSALEQDAKVLE AEQIAEDELTLSLSQLISN KEWQE
8002953181	*Dickeya solani* RNS 05.1.2A	Pseudomonadota	116/275 42.18%	MDKAQKLELAVAELNAEN TEAENEILLDVTCMVSNK EWQE
2559216572	*Dickeya* sp. NCPPB 3274	Pseudomonadota	116/275 42.18%	MDKAQELELAVAELNAEN TEAENEILLDVTCMVSNK EWQE
8044042700	*Pseudomonas coronafaciens* pv. *atropurpurea* ICMP 4451	Pseudomonadota	118/280 42.14%	MELQEKDVALEEQELAIA KSEELSEELVLSLAHHGS DKEWQE
2834855307	*Dickeya undicola* FVG10-MFV-A16	Pseudomonadota	115/276 41.67%	MDKAQELELAVAELNAEN AEAENEILLDVTCMVSNK EWQE
8064555015	*Yokenella regensburgei* NCTC 12131	Pseudomonadota	116/280 41.43%	MDKSQELELAVAELNAEN AEAENELLLDVTCLVSNK EWQE
2757616447	*Yokenella regensburgei* DSM 5079	Pseudomonadota	116/280 41.43%	MDKSQELELAVAELNAEN AEAENELLLDVTCLVSNK EWQE
8102243379	*Caballeronia* sp. LZ043	Pseudomonadota	115/278 41.37%	MIMNEQNQAVLENDVRQI DVEQVADDELTLALSHVV SNKEWQE

8025682312	*Caballeronia hypogeia* LZ043	Pseudomonadota	115/278 41.37%	MIMNEQNQAVLENDVRQIDVEQVADDELTLALSHVVSNKEWQE
2629162083	*Dickeya undicola* 2B12	Pseudomonadota	114/276 41.30%	MDKAQELELAVAELNAENAEAENEILLDVTCMVSNKEWQE
2773180829	*Xenorhabdus ehlersii* DSM 16337	Pseudomonadota	113/275 41.09%	MDKAQELEIAVAELNAENSDAENELLLDITGVVSNKEWQE
2839587921	*Xenorhabdus ishibashii* DSM 22670	Pseudomonadota	113/275 41.09%	MDKAQELEIAVAELNAENSDAENELLLDITGVVSNKEWQE
8025655193	*Caballeronia hypogeia* LZ032	Pseudomonadota	114/278 41.01%	MIMNEQNQAVLENDVRQIDVEQVTDDELTLALSHVVSNKEWQE
8102212807	*Caballeronia* sp. LZ032	Pseudomonadota	114/278 41.01%	MIMNEQNQAVLENDVRQIDVEQVTDDELTLALSHVVSNKEWQE
2769553685	*Pseudomonas* sp. 37 R 15	Pseudomonadota	103/276 37.32%	MDSQQATQLELDEQLVEVDLHAEVDDELVFAISAPTSNKEWQE
2769519119	*Pseudomonas* sp. 28 E 9	Pseudomonadota	103/276 37.32%	MDSQQATQLELDEQLVEVDLHAEVDDELVLAISAPTSNKEWQE

Table S3. HR-MS/MS fragmentation data for Arg-C Ultra fragment of unmodified StdA.

Ion	Observed *m/z*	Calculated *m/z*	Δ ppm	Sequence
b_3	287.0988	287.0986	0.0002	GND
b_4	402.1255	402.1256	0.0001	GNDD
b_5	515.2092	515.2096	0.0004	GNDDI
b_6	586.2464	586.2467	0.0003	GNDDIA
b_7	699.3309	699.3308	0.0001	GNDDIAL
b_8	770.3674	770.3679	0.0005	GNDDIALA
b_9	857.3995	857.3999	0.0004	GNDDIALAS
b_{10}	956.4703	956.4683	0.002	GNDDIALASV
b_{11}	1070.5123	1070.5113	0.001	GNDDIALASVN
b_{12}	1157.5332	1157.5433	0.0101	GNDDIALASVNS
b_{13}	1271.5896	1271.5862	0.0034	GNDDIALASVNSN
b_{14}	1399.6791	1399.6812	0.0021	GNDDIALASVNSNK
b_{15}	1528.7231	1528.7238	0.0007	GNDDIALASVNSNKE
b_{16}	1714.7986	1714.8031	0.0045	GNDDIALASVNSNKEW
y_2	276.1191	276.1190	0.0001	QE
y_3	462.1979	462.1983	0.0004	WQE
y_4	591.2402	591.2409	0.0007	EWQE
y_5	719.3354	719.3359	0.0005	KEWQE
y_6	833.3771	833.3788	0.0017	NKEWQE
y_7	920.4091	920.4108	0.0017	SNKEWQE
y_8	1034.4514	1034.4538	0.0024	NSNKEWQE
y_9	1133.5203	1133.5222	0.0019	VNSNKEWQE
y_{10}	1220.5522	1220.5542	0.002	SVNSNKEWQE
y_{11}	1291.5896	1291.5913	0.0017	ASVNSNKEWQE
y_{12}	1404.6770	1404.6754	0.0016	LASVNSNKEWQE
y_{13}	1475.7136	1475.7125	0.0011	ALASVNSNKEWQE
y_{14}	1588.7948	1588.7966	0.0018	IALASVNSNKEWQE
y_{15}	1703.8313	1703.8235	0.0078	DIALASVNSNKEWQE
y_{16}	1818.8472	1818.8504	0.0032	DDIALASVNSNKEWQE

Table S4. HR-MS/MS data for Arg-C Ultra fragment of StdA following the in vitro reaction with StdGH in the presence of ATP and Na_2S.

Ion	Observed m/z	Calculated m/z	Δ ppm	Sequence
b_3	287.0990	287.0986	0.0004	GND
b_4	402.1256	402.1256	0	GNDD
b_5	515.2098	515.2096	0.0002	GNDDI
b_6	586.2473	586.2467	0.0006	GNDDIA
b_7	699.3293	699.3308	0.0015	GNDDIAL
b_8	770.3698	770.3679	0.0019	GNDDIALA
b_9	857.4064	857.3999	0.0065	GNDDIALAS
b_{10}	956.4700	956.4683	0.0017	GNDDIALASV
b_{11}	1070.5110	1070.5113	0.0003	GNDDIALASVN
b_{12}	1157.5421	1157.5433	0.0012	GNDDIALASVNS
b_{14}	1415.6562	1415.6512	0.005	GNDDIALASVNSnK
b_{15}	1544.7006	1544.6938	0.0068	GNDDIALASVNSnKE
b_{16}	1730.782	1730.7731	0.0089	GNDDIALASVNSnKEW
y_2	276.1193	276.1190	0.0003	QE
y_3	462.1983	462.1983	0	WQE
y_4	591.2411	591.2409	0.0002	EWQE
y_5	719.3402	719.3359	0.0043	KEWQE
y_6	849.3528	849.3488	0.004	nKEWQE
y_7	936.3867	936.3808	0.0059	SnKEWQE
y_8	1050.4291	1050.4238	0.0053	NSnKEWQE
y_9	1149.4984	1149.4922	0.0062	VNSnKEWQE
y_{10}	1236.5306	1236.5242	0.0064	SVNSnKEWQE
y_{11}	1307.5674	1307.5613	0.0061	ASVNSnKEWQE
y_{12}	1420.6522	1420.6454	0.0068	LASVNSnKEWQE
y_{13}	1491.6895	1491.6825	0.007	ALASVNSnKEWQE
y_{14}	1604.7765	1604.7666	0.0099	IALASVNSnKEWQE

Table S5. HR-MS/MS data for Arg-C Ultra fragment of StdGH-modified StdA peptide following the in vitro reaction with StdF in the presence of carbamoyl phosphate and ATP. This species represents the carbamoylated full-length peptide.

Ion	Observed m/z	Calculated m/z	Δ ppm	Sequence
b_3	287.0988	287.0986	0.0002	GND
b_4	402.1251	402.1256	0.0005	GNDD
b_5	515.2084	515.2096	0.0012	GNDDI
b_6	586.2462	586.2467	0.0005	GNDDIA
b_7	699.3296	699.3308	0.0012	GNDDIAL
b_8	770.3666	770.3679	0.0013	GNDDIALA
b_9	857.3995	857.3999	0.0004	GNDDIALAS
b_{10}	956.4658	956.4683	0.0025	GNDDIALASV
b_{11}	1070.5096	1070.5113	0.0017	GNDDIALASVN
b_{12}	1157.5464	1157.5433	0.0031	GNDDIALASVNS
b_{13}	1287.5669	1287.5562	0.0107	GNDDIALASVNSn
b_{14}	1458.6632	1458.6570	0.0062	GNDDIALASVNSnk
y_2	276.119	276.1190	0	QE
y_3	462.1977	462.1983	0.0006	WQE
y_4	591.2402	591.2409	0.0007	EWQE
y_5	762.3392	762.3417	0.0025	kEWQE
y_8	1093.4354	1093.4296	0.0058	NSnkEWQE

Table S6. HR-MS/MS data for Arg-C Ultra fragment for cleaved StdGH-modified StdA peptide following the in vitro reaction with StdF in the presence of carbamoyl phosphate and ATP, and StdDE in the presence of iron and ascorbate.

Ion	Observed *m/z*	Calculated *m/z*	Δ ppm	Sequence
b_3	287.0995	287.0986	0.0009	GND
b_4	402.1265	402.1256	0.0009	GNDD
b_5	515.2108	515.2096	0.0012	GNDDI
b_6	586.2482	586.2467	0.0015	GNDDIA
b_7	699.3339	699.3308	0.0031	GNDDIAL
b_8	770.3704	770.3679	0.0025	GNDDIALA
b_9	857.4037	857.3999	0.0038	GNDDIALAS
b_{12}	1157.5503	1157.5433	0.007	GNDDIALASVNS
y_1	222.1092	222.1084	0.0008	k
y_2	352.1296	352.1213	0.0083	nk
y_3	439.1618	439.1534	0.0084	Snk
y_4	553.2064	553.1963	0.0101	NSnk
y_6	739.3060	739.2967	0.0093	SVNSnk
y_7	810.3428	810.3338	0.009	ASVNSnk
y_8	923.4227	923.4179	0.0048	LASVNSnk
y_9	994.4649	994.4550	0.0099	ALASVNSnk

Table S7. Plasmids and bacterial strains used in this study.

Plasmids	Purpose	Source
pRSFDuet-1_*stdH_stdG*	Kan^R, Coexpression of StdG and StdH	This study
pRSFDuet-1_*stdF*	Kan^R, Coexpression of StdG and StdGH	This study
pRSFDuet-1_*stdD_stdE*	Kan^R, Coexpression of StdD and StdE	This study
**Bacterial strains**	**Purpose**	**Source**
E. coli BL21 (DE3)	Coexpression of StdDE and expression of StdF	NEB
E. coli Rosetta2 (DE3) pLysS	CM^R, Coexpression of StdGH	NEB

Table S8. LC-MS ESI data for StdA following the in vitro reaction with StdGH in the presence of ATP and Na_2S.

Substrate	Enzyme	Product	Ion	Observed *m/z*	Calculated *m/z*	Δ ppm
StdA	No enzyme	StdA	$[M+10H]^{10+}$	665.8316	665.7290	0.1026
			$[M+9H]^{9+}$	739.7006	739.5878	0.1128
			$[M+8H]^{8+}$	832.0387	831.9113	0.1274
			$[M+7H]^{7+}$	950.7580	950.6129	0.1451
			$[M+6H]^{6+}$	1109.0518	1108.8817	0.1701
			$[M+5H]^{5+}$	1330.6643	1330.4480	0.2163
			$[M+4H]^{4+}$	1663.0825	1662.8225	0.26
StdA	StdGH	StdGH-modified StdA	$[M+10H]^{10+}$	667.4294	667.3240	0.1054
			$[M+9H]^{9+}$	741.4731	741.3600	0.1131
			$[M+8H]^{8+}$	834.0325	833.9050	0.1275
			$[M+7H]^{7+}$	953.0361	952.8914	0.1447
			$[M+6H]^{6+}$	1111.7092	1111.5400	0.1692
			$[M+5H]^{5+}$	1333.8506	1333.6480	0.2026

Table S9. LC-MS ESI Data for StdGH-modified StdA following in vitro reactions with (1) StdDE in the presence of iron and ascorbate and (2) StdF in the presence of carbamoyl phosphate and ATP.

Substrate	Enzyme	Product	Ion	Observed *m/z*	Calculated *m/z*	Δ ppm
StdGH-modified StdA	StdDE	StdGH-modified StdA	$[M+10H]^{10+}$	667.4294	667.3240	0.1054
			$[M+9H]^{9+}$	741.4731	741.3600	0.1131
			$[M+8H]^{8+}$	834.0325	833.9050	0.1275
			$[M+7H]^{7+}$	953.0361	952.8914	0.1447
			$[M+6H]^{6+}$	1111.7092	1111.5400	0.1692
			$[M+5H]^{5+}$	1333.8506	1333.6480	0.2026
StdGH-modified StdA	StdF	Full-length StdGHF-modified StdA	$[M+9H]^{9+}$	746.1476	746.1311	0.0165
			$[M+8H]^{8+}$	839.2907	839.2725	0.0182
			$[M+7H]^{7+}$	959.0450	959.0257	0.0193
			$[M+6H]^{6+}$	1118.7221	1118.6967	0.0254
			$[M+5H]^{5+}$	1342.2653	1342.2360	0.0293
StdGH-modified StdA	StdF	Cleaved StdGHF-modified StdA	$[M+9H]^{9+}$	682.5701	682.5533	0.0168
			$[M+8H]^{8+}$	767.7634	767.7475	0.0159
			$[M+7H]^{7+}$	877.3003	877.2829	0.0174
			$[M+6H]^{6+}$	1023.3502	1023.3300	0.0202
			$[M+5H]^{5+}$	1227.8215	1227.7960	0.0255

Table S10. LC-MS ESI Data for StdGHF-modified StdA following in vitro reactions with StdDE in the presence of iron and ascorbate.

Substrate	Enzyme	Product	Ion	Observed m/z	Calculated m/z	Δ ppm
Cleaved StdGHF-modified StdA	StdDE	Cleaved StdGHFDE-modified StdA (mono-hydroxylation)	$[M+9H]^{9+}$	684.3317	684.3433	0.0116
			$[M+8H]^{8+}$	769.7441	769.7612	0.0171
			$[M+7H]^{7+}$	879.5641	879.5943	0.0302
			$[M+6H]^{6+}$	1025.9902	1026.0150	0.0248
			$[M+5H]^{5+}$	1230.9912	1231.0180	0.0268
			$[M+4H]^{4+}$	1538.4871	1538.5225	0.0354
Cleaved StdGHF-modified StdA	StdDE	Cleaved StdGHFDE-modified StdA (bis-hydroxylation)	$[M+8H]^{8+}$	771.8662	771.7612	0.105
			$[M+7H]^{7+}$	881.9900	881.8700	0.12
			$[M+6H]^{6+}$	1028.8221	1028.6817	0.1404
			$[M+5H]^{5+}$	1234.3870	1234.2180	0.169
			$[M+4H]^{4+}$	1542.7346	1542.5225	0.2121
			$[M+3H]^{3+}$	2056.6452	2056.3633	0.2819

Table S11. HPLC retention times of Arg-C Ultra digested modified StdA products.

ArgC-Ultra cleaved product	Retention time (min)
Unmodified StdA	26.45
StdGH-modified StdA	17.65
StdGHF-modified StdA (full-length)	30.28
StdGHFDE-modified StdA	25.15

DNA and Amino Acid Sequences

StdA

Amino acid sequence

MSDNTAAVLSAIDSDKALRSAVIRHLATTHAHEFVGPLRAEFRGNDDIALASVNSNKEW
QE

DNA sequence

ATGTCCGACAACACCGCTGCGGTACTGTCCGCCATCGACAGCGACAAGGCGCTG
CGTTCGGCGGTCATCCGGCACCTCGCCACGACCCACGCCCACGAGTTCGTCGGT
CCGCTGCGCGCCGAGTTCCGCGGCAACGACGACATCGCCCTGGCGTCGGTCAAC
TCCAACAAGGAGTGGCAGGAGTAA

StdD (Multiple Cloning Site 1)

Amino acid sequence

MRTLSSGVTQALLLRDDREARTELLLRLLYSKSSADVVADALKDPSGRMCEATASVLS
TIDFHALEVERSARLRNLNRHLQRTCPPLWDCVEAETITPLVEAYADSDAFWQSAGRT
LFENFCLFAHRTVAGQSQLLADVFQLFGLVSRFSAPELIAPPEDVCACTTGIPGEGMDL
AKAAFSPWDDGGLIPPHVPDALQTESFRSAWRLVDERGRLPRSLDPDALGDPGDYQI
VVAAFPGRKVSAAALPLSGRCA

DNA sequence

TTGCGCACCCTTTCTTCCGGCGTGACGCAGGCACTCCTGTTGCGCGACGACAGAG
AGGCCCGTACGGAGTTGCTGCTCCGCCTGCTGTACTCCAAGAGCTCCGCGGACGT
GGTCGCCGATGCGCTGAAAGACCCCTCCGGCAGGATGTGCGAAGCAACGGCGTC
CGTTCTTTCCACCATCGATTTCCACGCCCTGGAGGTCGAGCGAAGCGCCCGTCTG
CGCAATCTGAACCGTCATCTGCAACGCACGTGCCCGCCTCTGTGGGATTGCGTCG
AAGCGGAAACGATCACTCCGCTCGTCGAGGCCTACGCGGACTCCGATGCTTTCTG
GCAGAGCGCCGGTCGCACCCTGTTCGAGAATTTCTGTCTGTTCGCCCACAGGACC
GTCGCCGGGCAGTCCCAGCTGCTCGCGGACGTCTTTCAGCTGTTCGGCCTCGTGA
GCCGCTTCTCCGCTCCCGAGTTGATCGCGCCTCCCGAGGACGTGTGTGCCTGCAC
GACCGGTATTCCGGGAGAGGGCATGGATCTGGCGAAGGCCGCCTTCTCCCCCTG
GGACGACGGCGGACTGATCCCCCCGCACGTTCCGGACGCCCTCCAGACCGAGTC
GTTCCGCTCTGCCTGGCGCCTCGTGGACGAGCGCGGGCGGCTGCCGCGCTCCCT
CGACCCCGACGCCCTCGGTGACCCCGGTGACTACCAGATCGTTGTCGCCGCCTTC
CCCGGCCGCAAGGTGTCCGCCGCGGCCCTTCCGCTCTCAGGCAGGTGCGCATGA

DNA sequence (E. coli optimized)

ATGAGGACATTATCAAGTGGAGTAACTCAAGCGTTGCTCCTACGCGATGACCGTGA
AGCTAGAACTGAGTTGCTGTTACGCTTGCTTTACAGCAAGTCGAGCGCCGATGTCG
TCGCCGACGCGCTGAAGGATCCGTCTGGTCGTATGTGCGAAGCTACGGCAAGCG
TTCTGAGTACCATCGATTTTCACGCTTTGGAAGTTGAACGTAGCGCGCGTTTGCGC
AACCTGAATCGTCATCTGCAACGTACCTGCCCGCCGCTGTGGGACTGTGTTGAGG
CCGAAACGATTACCCCGTTAGTAGAGGCGTATGCAGACAGCGATGCGTTCTGGCA
GTCGGCTGGCCGCACCCTGTTCGAGAACTTCTGCCTGTTCGCACACCGTACGGTT
GCTGGCCAGTCCCAGCTGTTGGCCGACGTGTTTCAACTGTTCGGCCTGGTGAGCC

GTTTTTCCGCGCCTGAGTTGATCGCTCCACCGGAGGATGTGTGCGCGTGCACCAC
CGGTATTCCGGGTGAAGGCATGGATCTGGCTAAAGCAGCGTTCTCTCCGTGGGAC
GACGGCGGTCTGATCCCGCCGCATGTTCCGGATGCACTGCAAACCGAGAGCTTTC
GTAGCGCGTGGCGTCTGGTTGATGAACGCGGTCGTCTCCCGCGTTCTCTGGATCC
AGACGCGCTGGGTGACCCGGGCGACTACCAGATTGTGGTGGCCGCGTTTCCGGG
TCGCAAAGTGTCAGCGGCAGCGCTGCCGCTGTCCGGTCGCTGTGCGTAA

StdE (Multiple Cloning Site 2)

Amino acid sequence

MTKEKKDPVGLLGFGLDWQYRDSYPQKMPDLCARYAGRLSHLSCVSLPSAADAQTF
VDECAGDLPVVHHLPGVAPAAPGGPNMELFTRLEAVSDVLGAVWTCEDIGLWSIGPY
PLPYFTPPLFEREIADHVVDGIRRMQEVSRYPFVPEIPSCTLAVGRMSLGEFFHRVTDG
ADCDLLLDVAHVFSYAVAVRQPYEDVLRSLPLDRVVEIHVAGGYIDPTFDNRYLDTHSH
AVTPAVIDLLQEAVAHAPRLRAVTYEIGVGLGADELDSDFERIENLLAEVGWTPSISRPS
GLATAGTA

DNA sequence

ATGACGAAAGAGAAGAAGGACCCCGTCGGCCTGCTCGGCTTCGGACTCGACTGG
CAGTACCGCGACAGCTACCCGCAGAAGATGCCGGACCTGTGCGCCCGGTACGCC
GGCCGGCTCAGCCATCTGTCCTGTGTCTCCCTGCCCAGCGCCGCCGACGCGCAG
ACCTTCGTGGACGAGTGCGCCGGCGACCTGCCTGTGGTGCACCACCTGCCGGGC
GTGGCACCCGCAGCGCCCGGCGGCCCCAACATGGAGCTGTTCACCCGCCTCGAG
GCGGTCAGCGACGTGCTGGGTGCCGTGTGGACCTGTGAGGACATCGGCCTGTGG
TCGATCGGCCCGTACCCGCTGCCGTACTTCACCCCTCCGCTCTTCGAGCGCGAGA
TCGCCGACCATGTCGTCGACGGCATACGCCGCATGCAGGAGGTCAGCCGTTACC
CCTTCGTGCCGGAGATCCCGTCCTGCACCCTCGCCGTGGGCCGTATGAGCCTCG
GCGAGTTCTTCCATCGCGTCACCGACGGCGCCGACTGCGACCTGCTGCTGGACG
TCGCGCATGTCTTCTCGTACGCCGTCGCCGTGCGGCAGCCCTACGAGGACGTGCT
CCGCTCGCTTCCGCTGGACCGGGTGGTGGAGATCCATGTGGCGGGCGGTTACAT
CGACCCGACCTTCGACAACCGGTACCTGGACACCCACAGCCACGCCGTGACCCC
GGCCGTCATCGACCTGCTCCAGGAGGCGGTCGCCCACGCGCCGCGGCTGCGCG
CCGTCACCTACGAGATCGGGGTGGGTCTCGGGGCCGACGAGCTGGACAGCGACT
TCGAGCGCATCGAGAACCTGCTGGCGGAGGTCGGGTGGACCCCGTCCATCTCCC
GTCCGTCCGGCCTGGCCACGGCAGGTACCGCATGA

DNA sequence (E. coli optimized)

ATGACAAAAGAAAAGAAAGATCCCGTAGGACTCTTGGGCTTCGGCCTCGACTGGC
AGTATCGCGATAGCTACCCGCAGAAAATGCCGGATCTTTGCGCGCGTTACGCTGG
CCGTCTGAGCCACCTGTCATGCGTTAGCCTGCCGTCTGCGGCGGATGCTCAAACC
TTTGTTGACGAGTGCGCAGGCGATCTGCCGGTTGTTCACCACCTGCCGGGTGTGG
CGCCAGCAGCGCCTGGTGGTCCGAATATGGAACTGTTCACGCGTCTGGAAGCGG
TCTCCGATGTTCTGGGCGCGGTTTGGACGTGCGAAGACATCGGCCTGTGGAGCAT
CGGCCCGTACCCGTTGCCGTACTTCACCCCACCGTTGTTTGAACGTGAAATCGCT
GACCACGTGGTGGATGGTATTCGCCGTATGCAAGAGGTGAGCCGTTACCCGTTTG
TCCCGGAGATCCCGTCTTGTACCCTGGCGGTTGGGCGTATGAGCCTGGGCGAATT

CTTTCATCGTGTTACCGACGGTGCTGACTGTGATTTGCTGCTGGACGTTGCGCACG
TGTTTAGCTATGCGGTGGCCGTACGCCAGCCGTACGAGGATGTTCTTAGATCGTT
GCCGTTGGACCGCGTAGTGGAAATTCATGTGGCGGGTGGCTATATTGATCCGACC
TTCGATAACCGTTATCTGGACACCCATTCCCACGCCGTGACTCCGGCAGTGATTGA
TTTACTGCAAGAGGCGGTCGCCCATGCACCGCGTCTGCGCGCAGTCACCTATGAG
ATCGGTGTTGGTCTGGGTGCTGACGAGCTGGACTCCGACTTCGAGCGCATTGAAA
ACCTGTTGGCCGAAGTTGGTTGGACTCCGTCGATCAGCCGTCCCAGCGGTCTGGC
TACCGCGGGCACGGCATAA

StdF **(Multiple Cloning Site 1)**

Amino acid sequence

MDPVHLPSVRPGHGRYRMTHAQQPASPPWSVLSPEPDRGAGLGEGVRVLVVSDGT
PAPDGDGMPDAHTERVAGLCRQLAGRAEIGYASAARLRARSLAADLRAMVTADGGW
DIVAMPWSSPERDAERLKVRRAFEEVARDPDMPLFVAAAGHDGPGRLRFPASCPSVL
AVGVGSDHGGPTAYCGTDGLGRKPQLLVPDARYATRCAAGPAPMRGTSAAVGIVAG
LAAVLAQRLGSDGRRVPSPLLRAALLASAGPDGTLVGTPLLRQPGTGAAEPFCFELPA
AAARLRLRLRTGADTVRIAAVAAGADEVVSGDTAPLWLPRAAELSVTWAADGTTRRTT
GAGWLVLDVASGAGRDTVITLECPEPVTAYVAVGGAEAVERIDPRLEKTDEPGVTAPA
WRAGTGRPVVLGLSASHDASACLLRDGSPQVAVQQERVTRRKHDGVGHLSSR
AAADYCLTSAGLTADDVDVFAFNAQPLLPGYVGLSVPSAAQSFDLFDPFDARTVYVSH
HLAHAFSAFWGSPFEEAVVVVCDGSGGSVLGTDDLLVDGPGLREYLARGPEDRLPRI
HVFSVYHFDRDGYRLLFRECADSFNVRVGSSSIGETYAAVSQYVFGDWQEGSGKLM
GLAPWGKARNAGPSLLEPGPHGLPRFRSDWKDTYRAPGSGGPMDHADLAARIQADL
EEALVARMRYAMSLVPGCRNLAYAGGVALNSVANDRIARESGADRFFVFPAASDAGV
SLGAAAAAHYRLTGSTRRRQVPYDDYLGHPYTQDDHDAAIGLVGDRVVTEPLELEAVA
DRIAAGDVVGWFQGGSEFGPRALGHRSVLADARSRDTWDFINAHIKFREDFRPLAPIV
PEEVAAEYFDLDEPSPHMLRVVPVREKYREQLAAVTHVDGTARVQTVSRDMNPRIHE
LLHLVGARTGFPVLVNTSLNRRGEPMIETPGQALDMLLGTRLSAMVLGDRVVRRAPEN
EAPLTLRSRLVLAPGVRLRWEQDCEGSRSWITGGAEPAGLELPRWAFDALSRAEPGR
ALGDYLPDCLVSAQGGTDTALAFLTALRARCLLVVTREAVHD

DNA sequence

GTGGACCCCGTCCATCTCCCGTCCGTCCGGCCTGGCCACGGCAGGTACCGCATG
ACACACGCACAGCAGCCCGCCTCGCCGCCGTGGTCCGTACTGAGCCCGGAACCC
GACCGCGGCGCCGGGCTCGGGGAAGGCGTACGTGTCCTGGTCGTCTCGGACGG
CACTCCCGCGCCCGACGGCGACGGGATGCCCGACGCGCACACGGAGCGGGTCG
CCGGACTGTGCCGGCAGCTGGCCGGCCGGGCGGAGATCGGTTACGCCTCGGCC
GCACGGCTGAGGGCCCGCAGCCTCGCCGCCGACCTGCGTGCGATGGTCACGGC
GGACGGCGGCTGGGACATCGTGGCCATGCCGTGGTCGTCGCCCGAGCGTGACG
CGGAACGGCTCAAGGTGCGGCGGGCCTTCGAGGAGGTGGCCCGGGACCCGGAC
ATGCCGCTGTTCGTCGCCGCTGCCGGACACGACGGACCGGGACGGCTCAGGTTC
CCCGCCTCCTGCCCCTCCGTGCTCGCCGTCGGGGTCGGCTCCGACCACGGCGGA
CCGACGGCCTACTGCGGCACGGACGGCCTGGGCCGCAAGCCTCAGCTGCTGGTC
CCGGACGCCCGCTACGCCACCCGCTGCGCGGCCGGCCCCGCTCCCATGCGCGG
CACCTCCGCCGCCGTCGGCATCGTCGCCGGACTCGCCGCCGTACTCGCACAGCG
GCTGGGGAGCGACGGACGCCGGGTGCCCTCGCCGCTGCTGCGTGCCGCGCTGC

TGGCCTCCGCCGGGCCGGACGGAACGCTGGTGGGCACGCCGCTGCTGCGGCAG
CCGGGGACCGGTGCCGCAGAACCGTTCTGCTTCGAGCTGCCCGCCGCAGCCGCC
CGGCTGCGGCTGCGCCTGAGAACCGGCGCGGACACCGTTCGGATCGCAGCCGTC
GCCGCGGGCGCGGACGAGGTCGTCAGCGGGGACACCGCCCCGTTGTGGCTGCC
GCGCGCCGCCGAACTCTCCGTGACATGGGCGGCGGACGGCACGACCCGGCGGA
CCACCGGCGCCGGGTGGCTGGTGCTCGATGTCGCATCCGGCGCCGGGCGTGAC
ACGGTGATCACCCTGGAGTGCCCGGAACCGGTCACCGCCTATGTCGCCGTCGGC
GGCGCCGAGGCCGTGGAGCGGATCGACCCTCGCCTCGAGAAGACCGACGAGCC
CGGCGTGACCGCTCCGGCGTGGCGGGCCGGCACCGGCCGTCCCGTCGTCCTCG
GCCTGTCGGCCAGCCATGACGCCTCCGCCTGCCTGCTGCGCGACGGCAGCCCGC
AGGTCGCCGTGCAGCAGGAGCGGGTCACCCGCCGGAAACACGACGGAGTCGGC
CACCTCTCCTCCCGTGCGGCCGCCGACTACTGTCTCACCTCGGCCGGCCTGACC
GCCGACGACGTGGACGTCTTCGCCTTCAACGCACAACCGCTGCTGCCTGGTTACG
TGGGGCTGTCGGTGCCGTCCGCGGCGCAGTCGTTCGACCTGTTCGACCCCTTCG
ACGCGCGCACCGTCTACGTCTCGCACCACCTCGCCCACGCCTTCTCCGCGTTCTG
GGGCTCGCCCTTCGAGGAGGCCGTGGTCGTCGTCTGCGACGGCTCCGGAGGCTC
GGTGCTCGGCACGGACGACCTCCTGGTGGACGGTCCCGGACTGCGGGAGTACCT
CGCGCGGGGGCCCGAGGACCGGCTGCCGCGCATCCACGTCTTCTCCGTCTACCA
CTTCGACCGGGACGGCTACCGGCTGCTGTTCCGCGAGTGCGCCGACTCCTTCAAC
GTCCGCGTCGGCTCCTCCTCGATCGGCGAGACCTATGCGGCCGTGAGCCAGTAC
GTCTTCGGCGACTGGCAGGAGGGCAGCGGCAAGCTGATGGGCCTCGCCCCCTGG
GGGAAGGCCCGCAACGCCGGCCCCAGCCTGCTGGAGCCCGGCCCGCACGGCCT
GCCCCGCTTCCGCAGCGACTGGAAGGACACCTATCGCGCCCCCGGCTCCGGCGG
CCCGATGGACCACGCCGATCTCGCGGCCCGGATCCAGGCCGACCTCGAAGAGGC
ACTGGTGGCTCGCATGCGGTACGCCATGTCTCTGGTGCCCGGCTGCCGCAACCTC
GCCTACGCGGGCGGCGTCGCGCTCAACTCCGTGGCCAACGACCGCATCGCCCGC
GAGAGCGGAGCCGACCGCTTCTTCGTGTTCCCGGCGGCGAGCGACGCCGGGGTC
TCCCTGGGCGCCGCCGCAGCCGCGCACTACCGGCTCACCGGCTCGACCCGGCG
CCGGCAGGTCCCCTACGACGACTACCTGGGACACCCGTACACCCAGGACGACCA
CGACGCGGCCATCGGGCTGGTCGGCGACCGGGTGGTCACCGAGCCGCTGGAGC
TGGAGGCCGTGGCGGACCGGATCGCCGCGGGCGACGTCGTCGGCTGGTTCCAG
GGCGGATCGGAGTTCGGGCCACGCGCCCTGGGCCACCGGTCGGTCCTCGCCGA
CGCCAGGAGCCGCGACACCTGGGACTTCATCAACGCGCACATCAAGTTCCGGGA
GGACTTCCGCCCACTGGCGCCCATCGTGCCCGAGGAGGTCGCCGCCGAGTACTT
CGACCTCGACGAGCCCTCTCCGCACATGCTGCGGGTGGTCCCGGTCCGCGAGAA
GTACCGTGAGCAGCTCGCTGCCGTCACCCACGTCGACGGCACCGCCCGGGTGCA
GACCGTCTCGCGGGACATGAACCCGCGCATCCACGAACTGCTCCATCTCGTCGGC
GCACGCACCGGTTTCCCCGTCCTGGTGAACACCTCGCTCAACCGCCGTGGCGAG
CCCATGATCGAAACGCCCGGCCAGGCACTCGACATGCTGTTGGGCACGCGGCTG
AGCGCCATGGTGCTGGGCGACCGTGTGGTCCGTCGCGCCCCCGAGAACGAGGCA
CCGCTCACCCTGCGCTCCCGGCTCGTCCTCGCCCCCGGCGTGCGGCTGCGGTGG
GAACAGGACTGCGAGGGCAGCCGTTCGTGGATCACCGGCGGCGCCGAACCGGC
CGGCCTCGAGCTGCCGAGGTGGGCGTTCGACGCCCTGTCCCGGGCCGAGCCCG
GCCGTGCCCTCGGCGACTACCTGCCGGACTGCCTGGTCTCCGCACAGGGCGGCA
CCGACACGGCGCTGGCCTTCCTGACCGCGCTCCGTGCCCGCTGCCTGCTGGTGG
TCACCCGGGAGGCAGTGCATGACTGA

DNA sequence (E. coli optimized)

ATGGATCCAGTCCACCTACCCTCAGTTAGGCCGGGTCATGGCCGTTACCGCATGA
CCCACGCACAACAGCCGGCGTCTCCGCCTTGGTCAGTACTGAGCCCGGAGCCGG
ATAGAGGCGCAGGCTTGGGTGAGGGGGTACGCGTTCTGGTCGTGTCCGACGGTA
CTCCGGCTCCGGACGGCGATGGTATGCCGGACGCGCACACCGAACGGGTGGCTG
GCCTGTGTCGTCAACTGGCTGGCCGCGCGGAGATCGGTTATGCCAGCGCGGCTA
GGCTGCGTGCGCGCTCACTGGCGGCTGATCTGCGTGCTATGGTTACGGCTGACG
GCGGTTGGGACATCGTGGCTATGCCGTGGAGCTCTCCGGAACGGGACGCCGAAC
GGCTTAAGGTCCGTCGTGCATTCGAAGAGGTGGCGCGTGACCCGGATATGCCTCT
GTTTGTGGCGGCTGCCGGTCACGATGGTCCGGGCAGATTGCGCTTCCCGGCGTC
CTGTCCGTCTGTACTGGCGGTTGGTGTGGGCAGCGACCACGGCGGCCCGACCGC
GTACTGCGGTACCGACGGCCTTGGCCGCAAGCCGCAACTGCTGGTGCCGGATGC
ACGCTACGCTACCCGTTGCGCGGCGGGTCCGGCGCCGATGAGAGGTACATCTGC
GGCGGTCGGCATTGTGGCAGGGCTGGCGGCGGTGTTAGCTCAGCGTCTCGGTAG
CGATGGCCGTCGCGTCCCGTCACCGCTGCTACGTGCAGCTTTATTGGCGTCTGCT
GGTCCGGATGGTACGCTGGTGGGTACGCCGCTATTACGTCAACCGGGTACTGGTG
CCGCGGAACCTTTCTGCTTTGAGCTGCCAGCGGCGGCAGCTCGTCTGAGGCTTCG
GCTGCGCACCGGAGCCGACACCGTTCGTATCGCCGCTGTGGCAGCCGGCGCGGA
CGAAGTTGTTTCGGGCGACACCGCACCACTGTGGCTGCCGCGAGCTGCCGAACT
ATCTGTTACCTGGGCAGCCGACGGCACGACCCGCCGTACGACCGGTGCAGGCTG
GCTGGTCCTGGACGTGGCCTCTGGTGCCGGACGCGATACCGTTATTACCCTGGAG
TGCCCGGAACCGGTTACCGCATACGTGGCGGTGGGGGGCGCCGAAGCTGTGGAA
CGCATCGATCCGAGGCTGGAGAAAACCGACGAGCCGGGCGTTACAGCGCCAGCG
TGGCGTGCGGGCACCGGCCGCCCAGTTGTCTTGGGTTTGTCTGCCAGCCACGAC
GCGAGCGCATGTCTGCTGAGAGACGGCAGCCCGCAGGTCGCCGTACAACAAGAA
CGTGTGACACGTCGTAAACATGACGGCGTCGGGCACCTGTCCAGCCGCGCAGCG
GCAGACTACTGCCTGACCAGTGCTGGCCTGACCGCAGACGACGTGGATGTTTTCG
CCTTTAACGCACAACCGCTCCTGCCAGGCTATGTTGGTCTGTCCGTTCCATCGGCC
GCGCAAAGCTTCGACCTTTTCGACCCGTTTGACGCGCGCACGGTTTACGTGAGCC
ATCATCTTGCGCACGCATTCTCAGCATTCTGGGGTTCCCCGTTCGAGGAGGCAGTT
GTTGTCGTATGTGATGGCAGCGGTGGTAGCGTGCTGGGTACCGATGACCTGCTGG
TAGATGGTCCAGGCCTGCGTGAGTATCTGGCGCGTGGCCCGGAGGACCGTCTGC
CGAGAATCCACGTTTTTAGCGTTTATCATTTTGATCGTGACGGCTACAGGCTGCTG
TTCCGCGAATGCGCAGACAGCTTTAATGTTCGCGTGGGCAGCTCAAGCATTGGTG
AAACCTACGCGGCAGTGAGCCAGTATGTCTTCGGCGATTGGCAGGAGGGTTCTGG
CAAGCTGATGGGTCTGGCGCCGTGGGGTAAAGCACGCAACGCTGGTCCGTCTCT
GTTGGAGCCGGGCCCGCACGGTCTCCCGAGATTCCGTAGCGATTGGAAAGATAC
GTACCGTGCGCCGGGTAGCGGTGGTCCTATGGATCACGCCGACCTGGCGGCGCG
TATCCAGGCAGACCTAGAGGAGGCGCTGGTCGCACGTATGCGTTACGCCATGAGC
CTTGTTCCGGGGTGCCGTAACCTGGCCTACGCAGGCGGTGTTGCTTTGAACAGCG
TGGCTAATGATCGCATCGCACGTGAGAGCGGTGCGGACCGCTTCTTTGTTTTCCC
GGCGGCTAGCGACGCAGGTGTCTCCTTGGGCGCGGCTGCGGCGGCGCATTATCG
TCTGACCGGTAGCACCAGACGTCGTCAAGTTCCGTATGATGATTATCTGGGACACC
CGTATACCCAGGATGATCATGACGCTGCGATTGGTCTGGTGGGCGATCGCGTGGT
GACCGAACCGTTGGAGCTGGAGGCGGTTGCGGACCGTATTGCAGCCGGTGATGT

GGTTGGTTGGTTTCAGGGTGGATCCGAATTTGGTCCGCGCGCGTTGGGCCACCGT
AGCGTACTGGCGGACGCGCGTTCTCGTGACACTTGGGATTTTATCAACGCACACA
TTAAATTCCGTGAAGATTTTCGTCCGCTGGCCCCTATTGTCCCGGAAGAGGTTGCC
GCGGAATATTTCGATTTAGATGAACCGTCCCCGCATATGTTGAGAGTGGTTCCGGT
GCGTGAGAAGTACCGCGAGCAGCTCGCGGCTGTGACCCATGTTGATGGGACGGC
TCGTGTTCAGACCGTTAGTCGCGACATGAATCCGCGAATCCACGAACTGTTGCAC
CTGGTAGGTGCGCGTACCGGTTTTCCGGTGCTGGTCAACACCAGCCTGAATCGAC
GTGGTGAACCCATGATTGAAACCCCGGGTCAGGCTTTGGATATGCTGCTGGGTAC
GCGTCTGTCTGCCATGGTTTTAGGCGATCGTGTCGTGCGTCGTGCTCCAGAGAAC
GAAGCACCGTTGACCCTGCGTAGCCGACTGGTTCTCGCGCCGGGAGTACGTCTAC
GCTGGGAACAGGATTGCGAAGGTTCCCGTAGCTGGATCACCGGCGGTGCGGAAC
CGGCTGGCTTGGAGCTGCCGCGTTGGGCATTTGATGCTCTATCCAGAGCTGAGCC
GGGCAGGGCGTTGGGCGATTACTTGCCGGACTGTCTGGTTTCGGCTCAAGGTGG
CACTGACACCGCGTTAGCGTTCCTGACCGCACTGCGGGCGCGCTGCCTCTTGGTT
GTGACGCGCGAGGCAGTGCATGACTAA

StdG (Multiple Cloning Site 1)

Amino acid sequence

MTDLDDRMTDLDDTPRTHALAWLENTTDDATMLPCGTSHRSVPAADAVAKVWPLRH
RLGISRVTDLTPLDVVGIPVFSVTRPQARTGQITLCQGKGNTPVEALASALFEAVERHC
GSLARPTLTARPTELRAAGRTHLTAADLGLAEPVPDQPIEWIQGRDSRTGSPVLLPAAE
VIFPYTAPPGCLRPVRPSTTGLASGATLSEAVLHALYEVVERDATSRYLHGGPGRLVAL
DTVTGSAETELLRKYARAEIDTVVIDLTHTTVLPTFAVFLSDPGAAQAHLAVSGFGTHP
NAGVALRRALTEASQARATAIQGSREDLDRVAEVYRADPAEQRAAFLHRALQAKAEG
VTDMSTLPALPPRSTRDTLRHVAALLEEGGYDRVVHTDLTVPELGLPVAHVAVPGMVD
SVVEPLRSHRVAHQHVQA

DNA sequence

ATGACTGACCTGGACGACCGTATGACCGACCTGGACGACACGCCGCGCACGCAC
GCTCTGGCGTGGCTGGAGAACACGACCGACGACGCCACCATGCTGCCGTGCGGC
ACCTCGCACCGGTCGGTGCCTGCGGCCGATGCCGTCGCCAAGGTGTGGCCGCTG
CGCCACCGCCTCGGCATCAGCCGCGTCACGGACCTCACCCCGCTCGACGTCGTG
GGCATCCCCGTCTTCAGCGTGACCCGGCCTCAGGCCCGCACCGGGCAGATCACT
CTCTGCCAGGGAAAGGGCAACACACCTGTGGAAGCCCTCGCCTCCGCGCTGTTC
GAGGCGGTGGAGCGGCACTGCGGTTCCCTGGCCCGCCCCACACTGACGGCCCG
GCCGACCGAGCTGCGGGCCGCCGGCCGCACCCACCTGACCGCGGCGGACCTGG
GCCTGGCCGAGCCTGTGCCCGATCAGCCGATCGAGTGGATCCAGGGCCGGGACA
GCAGGACCGGCAGCCCCGTGCTGCTGCCCGCCGCCGAGGTGATCTTCCCGTACA
CCGCGCCGCCCGGCTGTCTGCGGCCGGTACGCCCGAGCACCACGGGCCTGGCC
TCCGGCGCCACGCTGAGCGAGGCGGTGCTGCATGCGCTGTACGAGGTCGTCGAA
CGCGACGCCACCTCGCGGTACCTGCACGGCGGGCCGGGCCGGCTGGTCGCCCT
CGACACGGTGACCGGATCCGCCGAGACGGAACTGCTGCGCAAGTACGCCAGGGC
CGAGATCGACACGGTCGTCATCGACCTCACCCACACCACTGTGCTGCCCACGTTC
GCGGTGTTCCTGTCCGACCCGGGTGCCGCCCAGGCCCACCTGGCGGTGTCCGGT
TTCGGTACACACCCCAACGCGGGCGTCGCCCTGCGCCGCGCCCTGACGGAGGCC
TCGCAGGCCCGTGCCACCGCCATCCAGGGCAGCCGGGAGGACCTCGACCGCGT

CGCCGAGGTCTACCGCGCCGACCCCGCCGAGCAGCGGGCTGCGTTCCTGCACCG
CGCCCTGCAGGCGAAGGCGGAAGGCGTCACCGACATGAGCACGCTCCCCGCCCT
GCCGCCGCGGTCGACACGCGACACACTGCGGCACGTGGCCGCGCTGCTGGAAG
AAGGCGGCTACGACCGGGTCGTGCACACCGATCTGACCGTCCCGGAACTCGGTC
TGCCCGTCGCCCATGTGGCGGTGCCCGGCATGGTCGACTCCGTCGTGGAACCCC
TCAGGAGCCATCGTGTGGCCCATCAGCACGTCCAGGCTTAG

DNA sequence (E. coli optimized)
ATGACTGACCTAGATGATAGGATGACAGATCTGGACGATACCCCGCGTACCCATG
CACTGGCATGGCTGGAGAACACCACCGATGATGCAACCATGCTGCCGTGTGGTAC
AAGCCATCGTTCGGTGCCGGCGGCCGACGCGGTCGCGAAGGTTTGGCCGCTGCG
CCATCGTCTGGGTATTAGCCGCGTGACCGATCTAACCCCGCTGGACGTTGTTGGT
ATCCCGGTTTTCAGCGTTACCCGTCCGCAGGCGCGCACGGGCCAAATTACCCTTT
GCCAGGGCAAAGGTAATACCCCGGTAGAGGCGCTGGCATCTGCTTTGTTTGAAGC
GGTCGAGCGTCACTGCGGTAGCCTGGCGCGTCCGACTTTGACTGCAAGACCAACA
GAACTGCGTGCGGCAGGTCGTACGCACTTGACGGCTGCGGATCTGGGCTTGGCG
GAGCCGGTGCCGGATCAACCGATTGAATGGATTCAAGGTCGCGACTCCAGGACG
GGTTCCCCAGTTCTGCTCCCAGCTGCGGAGGTCATCTTCCCGTATACCGCCCCTC
CTGGGTGCTTGCGCCCAGTGCGTCCGAGCACCACCGGACTGGCTTCTGGTGCCA
CCTTATCTGAAGCCGTGCTTCACGCCCTGTACGAAGTTGTGGAGAGAGATGCCAC
CAGCCGCTACCTGCATGGCGGTCCGGGCCGCCTGGTTGCGTTGGACACGGTTAC
CGGCTCCGCAGAGACAGAACTGTTGCGCAAGTACGCACGTGCCGAGATCGACAC
CGTGGTGATCGACCTGACCCACACCACCGTGCTGCCGACGTTTGCAGTGTTCCTG
AGCGACCCGGGTGCGGCGCAGGCGCACCTGGCTGTCAGCGGTTTCGGCACGCAT
CCGAACGCAGGCGTTGCTCTCCGTCGTGCGCTTACTGAGGCCTCGCAAGCTCGTG
CGACCGCGATCCAAGGTAGTCGGGAAGATCTCGACCGTGTTGCGGAAGTTTATCG
TGCGGACCCGGCGGAGCAGCGTGCGGCGTTTCTGCACCGCGCGCTGCAGGCGA
AAGCAGAGGGTGTCACCGACATGAGCACCCTGCCGGCGTTGCCACCGCGTTCCA
CTCGTGACACCCTGCGCCACGTGGCAGCGTTACTGGAGGAAGGCGGTTATGATC
GTGTGGTGCACACCGATTTGACCGTTCCGGAACTGGGCCTGCCGGTCGCGCACG
TTGCCGTCCCGGGCATGGTCGATAGCGTGGTTGAACCGCTGCGTAGCCATCGTGT
TGCTCACCAGCATGTACAGGCATAA

StdH (Multiple Cloning Site 2)
Amino acid sequence
MWPISTSRLRPIVFGAASVRPLDPALLAAVDLRPPVRRGDLLPLLDGDPGTVVLVDGLF
GGTMAVTPTECRQLLDAGWTVVGCSSMGALRAADLWPLGAIGIGDIFTLYRLGTLTSD
ADVAVALDPDDGHREVTVCTVHVRAVLAAAVEAGLIAPAQRARLARAAEGIHWSQRS
WTACRAVWSELGVAPTVLSSLLRLGREPRLHPKVRDAETCLRAVLAGDWLGDGATDT
RRCPACQKPLTAGMLFCSVCVSEAHETCL

DNA sequence
GTGTGGCCCATCAGCACGTCCAGGCTTAGGCCCATCGTCTTCGGCGCGGCGTCG
GTACGTCCCCTCGACCCCGCGCTGCTGGCAGCGGTCGACCTGCGGCCTCCGGTC
CGGCGCGGGGACCTGCTTCCGCTGCTGGACGGCGATCCCGGCACCGTGGTCCTC

GTCGACGGACTGTTCGGCGGCACGATGGCCGTCACCCCGACGGAGTGCCGTCAG
CTGCTCGACGCCGGCTGGACGGTGGTCGGCTGCTCCAGCATGGGCGCCCTGCGC
GCCGCCGACCTGTGGCCGCTCGGCGCGATCGGCATCGGTGACATCTTCACCCTG
TACCGGCTGGGCACGCTGACCTCCGACGCGGACGTCGCCGTCGCCCTGGACCCG
GACGACGGCCATCGGGAAGTGACCGTGTGCACGGTGCACGTCCGGGCCGTGCTC
GCGGCCGCCGTCGAGGCGGGTCTCATCGCCCCCGCCCAGCGGGCCCGGCTCGC
CCGTGCCGCCGAGGGCATCCACTGGTCCCAGCGGTCCTGGACGGCCTGCCGGG
CGGTGTGGAGCGAGCTGGGAGTCGCCCCCACGGTGCTCTCCTCACTTCTGCGGC
TCGGCAGGGAACCCCGGCTGCACCCCAAGGTGCGCGACGCCGAGACCTGCCTGC
GCGCTGTCCTGGCCGGCGACTGGCTCGGCGACGGGGCCACGGACACACGCCGC
TGTCCGGCCTGCCAGAAACCACTGACGGCAGGAATGCTGTTCTGTTCCGTCTGCG
TCTCCGAGGCGCACGAGACCTGCCTGTGA

DNA sequence (E. coli optimized)
ATGGTATGGCCCATAAGTACATCAAGACTAAGGCCGATTGTGTTCGGCGCTGCGA
GCGTTCGTCCGTTGGACCCGGCGTTGCTGGCTGCCGTTGATCTGCGCCCACCGG
TTCGCAGAGGCGACCTGCTGCCGCTCTTGGACGGCGACCCGGGTACTGTTGTCCT
GGTAGACGGCTTGTTTGGCGGTACCATGGCAGTCACCCCGACCGAATGTCGTCAG
CTGCTGGACGCGGGTTGGACGGTGGTTGGGTGCAGCTCCATGGGTGCTCTGCGC
GCTGCGGATCTGTGGCCTCTGGGTGCCATCGGCATCGGTGACATCTTTACCCTGT
ACCGCTTGGGTACGCTGACTAGCGATGCGGACGTTGCCGTGGCCCTGGATCCGG
ATGATGGTCATCGTGAGGTTACCGTCTGCACCGTTCACGTTCGGGCGGTGCTCGC
GGCTGCGGTGGAAGCAGGTCTGATTGCACCGGCACAACGTGCACGTTTAGCGCG
CGCAGCGGAAGGTATTCATTGGTCGCAACGTAGCTGGACCGCGTGCCGTGCCGT
GTGGTCTGAGCTGGGCGTGGCGCCAACCGTTTTAAGCTCTCTGCTGCGTTTGGGC
CGTGAGCCGCGTCTTCACCCGAAGGTGCGTGATGCAGAGACATGCCTGCGTGCG
GTCCTAGCTGGCGACTGGCTGGGCGACGGTGCTACCGATACCCGTCGCTGTCCG
GCGTGCCAGAAACCGTTGACCGCGGGTATGCTGTTCTGCTCCGTGTGTGTTAGCG
AGGCGCACGAAACGTGCCTGTAA
